# Correspondence of large-scale functional brain network decline across aging mice and humans

**DOI:** 10.1073/pnas.2527522123

**Published:** 2026-03-27

**Authors:** Ezra Winter-Nelson, Eyal Bergmann, Micaela Y. Chan, Gabriella Vill, Liang Han, Ziwei Zhang, Alexandra Kavushansky, Irit Dolgopyat, Jad Asleh, Jennifer D. Whitesell, Itamar Kahn, Gagan S. Wig

**Affiliations:** ^a^Center for Vital Longevity, The University of Texas at Dallas, Dallas, TX 75235; ^b^Department of Psychology, School of Behavioral and Brain Sciences, The University of Texas at Dallas, Dallas, TX 75080; ^c^Department of Neuroscience, Rappaport Faculty of Medicine, Technion—Israel Institute of Technology, Haifa, Israel 3525433; ^d^Zuckerman Mind Brain Behavior Institute and Department of Neuroscience, Columbia University, New York, NY; ^e^Allen Institute for Brain Science, Seattle, WA 98109; ^f^Department of Psychiatry, The University of Texas Southwestern Medical Center, Dallas, TX 75390

**Keywords:** brain networks, system segregation, aging, resting-state correlations, mouse

## Abstract

Human aging is marked by progressive reorganization of large-scale functional brain networks; these brain network changes have been linked to cognitive decline and disease vulnerability. Conversely, while mice have served as powerful models for understanding the molecular and cellular changes that occur over the lifespan, an absence of precise characterization of age-related changes in large-scale functional brain network organization has limited cross-species translational insights. Here, using densely sampled resting-state functional MRI data acquired cross-sectionally and longitudinally in awake mice over a broad range of adulthood (n = 82; 3 to 20 mo), we describe organizational features and age-related alterations of the mouse’s functional connectome. Mouse resting-state functional connectivity recapitulates known functional circuits, demonstrating the organizational validity of these signals. Graph theoretic analysis applied to functional connectivity reveals that mice exhibit modular architectures of functional brain network organization and that increasing age is associated with decreasing system segregation, indicative of network dedifferentiation analogous to observations in humans. Notably, mouse resting-state brain networks are more segregated than those of humans [determined using data from the Human Connectome Project and its developmental- and aging-counterparts (n = 1,179; 18 to 90 y)], attributable to mice exhibiting a diminished contribution of long-range functional relationships that integrate distributed systems. Mice also exhibit slower rates of age-related decline in brain network organization relative to humans, highlighting important species differences in functional brain network organization and trajectories of brain network aging. These findings establish a model of large-scale functional brain network aging in mice and provide a translational bridge across species and spatial scales of analysis.

## Significance

Human aging is accompanied by changes in large-scale functional brain network organization, which have important consequences for cognition and brain disease. Using awake functional MRI data acquired in mice across a range of adulthood, we demonstrate that aging mice exhibit alterations in brain network organization analogous to those in humans, particularly a loss in functional differentiation. In addition, there exist species-specific differences in network architecture and aging trajectories. These observations establish the mouse as a promising model for investigating the factors that confer resilience and vulnerability to age-related brain network decline and elucidating the cellular and molecular mechanisms of large-scale brain network changes. This meso-scale description of age-related changes in mouse brain networks provides a translational platform bridging species and organizational scales.

Human brain function is supported by the interactions of distributed large-scale brain networks (1, 2) and age-related cognitive decline is linked to a breakdown in structural and functional brain network organization (3, 4). Aging-related changes in functional brain network organization have been extensively mapped during resting wakefulness using functional MRI (5, 6). During adulthood, increasing age is associated with decreasing system segregation (7–10), a measure which quantifies the degree to which an individual’s brain network contains functionally distinct, or modular, brain systems (7, 11, 12). These changes indicate a progressive loss of the brain network’s functional specialization [i.e., a form of “dedifferentiation” at the network level; (12, 13)]. In keeping with this interpretation, lower system segregation is associated with worse memory ability (7, 10) and executive function (14, 15) in healthy individuals, and greater cognitive dysfunction among patients with Alzheimer’s Disease (AD) dementia (16–18).

Despite the growing body of work documenting changes in resting-state network organization across human adulthood, there is limited understanding of the cellular and molecular mechanisms that underlie age-related changes in brain network organization, and why some individuals are more susceptible to age-related brain network decline than others. Filling in these knowledge gaps is a critical step toward pinpointing genetic and environmental sources of brain network vulnerability and resilience (e.g., refs. 16 and 19) and for evaluating interventional and therapeutic strategies to modify trajectories of neurocognitive aging and AD. The development of nonhuman animal models of large-scale brain network aging would provide an important platform with which to accelerate progress toward these goals, analogous to that which has been achieved with the development of blood- (20) and tissue-based (21, 22) biomarkers of aging and health in other biological systems.

The mouse (*Mus musculus*) has been an important animal model for characterizing and studying processes of brain aging at behavioral (23–25) and neuronal levels (26–28), and for translational work in models of AD (29). However, an interareal, or “meso-scale,” description of aging brain function is lacking in mice. Establishing this scale of description would not only facilitate interactionist pursuits with the human research on brain network aging (30), but could also provide a mechanistic bridge between the microscale processes and behavioral outcomes in aging mice and animal models of age-related disease (e.g., refs. 31–33).

Previous work has demonstrated that resting state fMRI captures analogous aspects of neuronal signal in humans and mice (34–36), and that defining properties of resting-state networks are similarly conserved across species, including presence of strong functional connectivity among homotopic regions (37–41), stability of individual features (42), correspondence to structural connectivity (43, 44), modular organization (45), and relevance to task performance (42). While these observations have largely been made in young adult (YA) mice (e.g., at 3 mo of age), and primarily under anesthesia [cf. (34, 42, 46)], they indicate that the measurement of resting-state signals across distributed brain regions is a viable approach for investigating aging-related alterations of the mouse large-scale functional brain network architecture.

Here we evaluate whether mice exhibit age-related differences and changes in large-scale functional brain network organization across a large phase of adulthood, analogous to what is observed in aging human individuals. Importantly, this scale of analysis does not depend on establishing functional homologies between specific brain areas, functional systems, or cognitive domains across species, but rather is aimed at determining whether large-scale network architectures exhibit similar aging-accompanied alterations, regardless of the specific processing operations of their constituent components. To accomplish this goal, we leverage densely sampled cross-sectional and longitudinal awake passive mouse fMRI datasets spanning a wide range of adulthood (3 to 20 mo) and characterize trajectories of their functional brain network organization by adopting and further developing methods from human lifespan brain network analysis. This approach enables a cross-species comparison of large-scale functional brain network aging between mice and humans. We describe striking similarities in the dedifferentiation of functional brain network organization over the course of adulthood, and aspects where properties of brain network organization and their aging-related trajectories differ across species. In doing so, we establish an imaging-based biomarker of brain network function and organization that is accessible in the mouse and has direct links with observations in the neuroscience of human aging.

## Results

### Validation and Specificity of Resting-State Functional Correlations (RSFC) in Awake Mice.

We first determined whether awake resting-state fMRI signals contain functionally meaningful information in mice by evaluating the presence and dissociation of known functional circuits in individual animals [3 to 4 mo old, corresponding to early adulthood (47, 48); n = 31].

**Fig. 1.**
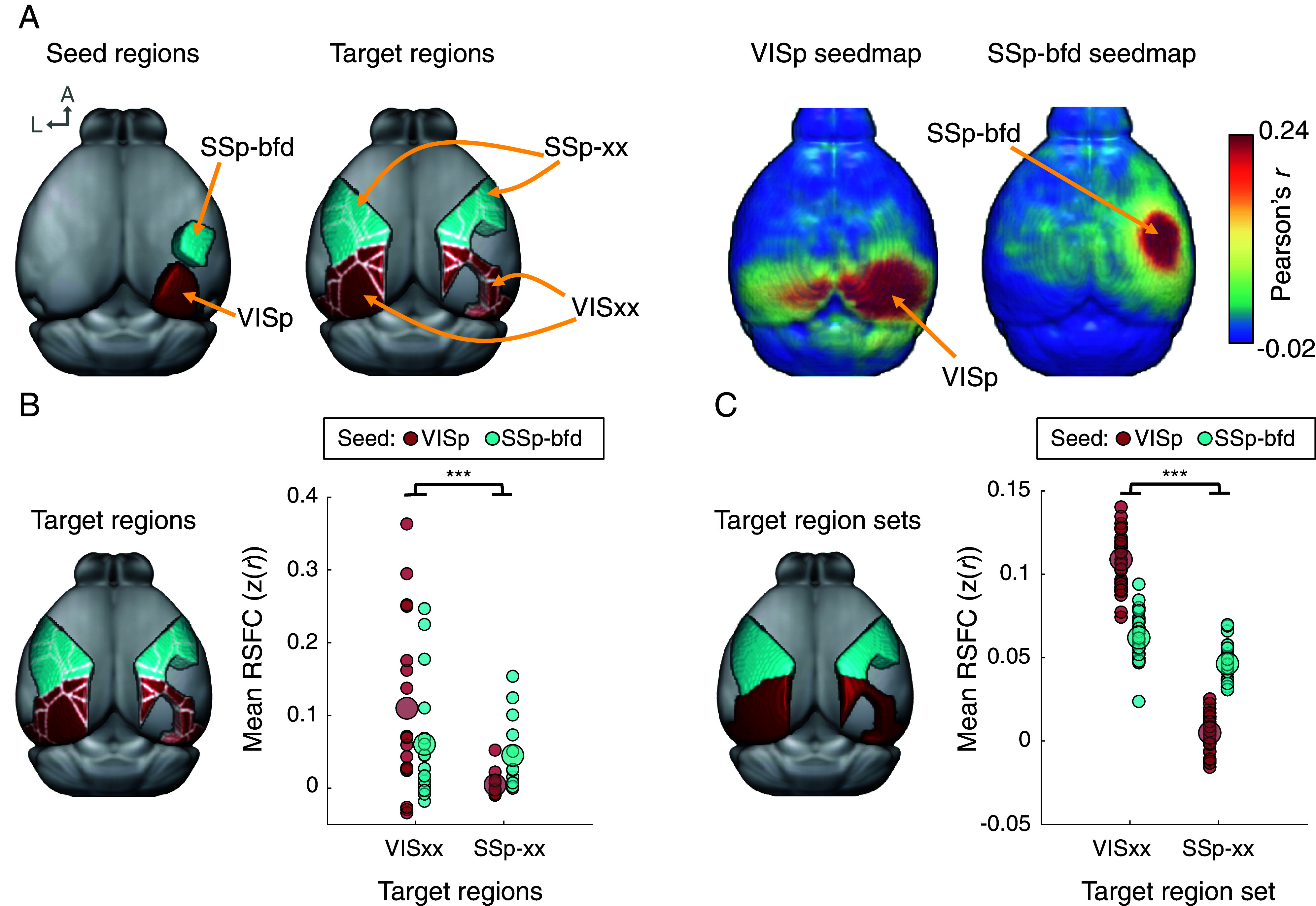
Awake resting-state signals recapitulate known dissociations of functional circuits among sensory regions in mice. (*A*) RSFC seedmaps were compared for two neighboring cortical regions to test the validity and specificity of functional connections. From *Left* to *Right*: right hemisphere primary visual cortex (VISp) and barrel cortex (SSp-bfd) parcels were used as RSFC seeds. Target regions were defined as CCFv3 parcels labeled as vision-related (VISxx; red) or primary somatosensory (SSp-xx; blue). Seed-correlation maps depict strengths of correlations across the cortex for each of the two seed regions. Despite the proximity of the VISp and SSp-bfd seeds, RSFC seedmaps for the two regions are topographically distinct and differ in their extent of contralateral (homotopic) functional connectivity. (*B*) Functional connectivity between seed and target regions was quantified by extracting group-average seedmap correlation values for each target region, to test whether VISxx targets are preferentially correlated with the VISp seed region, and whether SSp-xx targets are preferentially correlated with the SSp-bfd seed region. Each dot in (*B*) corresponds to a target region, where the dot’s value reflects the mean correlation between that target region and a given seed region. The brain image depicts the target regions used to extract mean correlations (i.e., parcels). (*C*) Specificity of VISp and SSp-bfd functional connectivity is present across individual mice. The brain image depicts the two target region sets used to extract mean correlations. When evaluated as a set of functionally related regions across mice, the VISp seed exhibits stronger RSFC with the VISxx target region set compared to the SSp-bfd seed. Conversely, the SSp-bfd seed exhibits stronger RSFC with the SSp-xx target region set compared to the VISp seed. Each dot corresponds to an individual mouse, where values reflect the mean correlation between each target region set and a given seed region. In (*B* and *C*), larger dots represent group means, as a visual aid. ***P < 0.001.

Appropriate node definition is a critical prerequisite for brain network analysis (49, 50). In the context of large-scale brain networks, nodes should represent functionally distinct areal units of the brain. In the present work, we define nodes using the Allen Institute’s Common Coordinate Framework [CCFv3; (51)], which partitions the mouse brain into functionally independent cortical and subcortical regions based on histology, gene expression, and axonal connectivity. Using the CCFv3 areal parcellation and its corresponding labels, we measured the RSFC among regions sharing similar functions and compared them to RSFC among other sets of functionally related regions to test for specificity. Mirroring validation work in other species (e.g., ref. 52), RSFC patterns were measured in neighboring cortical seed regions corresponding to primary unimodal areas: primary visual cortex (CCFv3 label: VISp) and primary somatosensory barrel cortex (CCFv3 label: SSp-bfd), which is involved with processing information from the whiskers. RSFC maps for these seed regions were compared both at the group average level and within individual mice; group-average seed-based correlation maps reveal distinct RSFC topographies for each seed location (Fig. 1*A*). As the sets of regions that share functional similarities with these seed regions are well defined (visual regions, denoted as VISxx and comprising CCFv3 parcels with the label prefix “VIS,” and primary somatosensory regions, denoted as SSp-xx and comprising CCFv3 parcels with the label prefix “SSp”), we evaluated specificity of RSFC by comparing correlations between each seed region and these two sets of target regions. We tested whether visual regions are preferentially correlated with VISp relative to SSp-bfd and whether primary somatosensory regions are preferentially correlated with SSp-bfd relative to VISp. Further, we evaluated specificity of RSFC for these relationships at two levels: by examining RSFC between the seed regions and each of the target regions (group-level analysis) as well as RSFC between the seed regions and each of the two aggregated target region sets (all visual vs. all somatosensory regions, individual-level analysis).

First, group-average RSFC seedmap values were extracted for every parcel labeled as “VIS” or “SSp” in the CCFv3, yielding four sets of connectivity groupings (VISp to VISxx, VISp to SSp-xx, SSp-bfd to VISxx, and SSp-bfd to SSp-xx). The visual regions predominantly exhibit greater functional connectivity with VISp, and somatosensory regions predominantly exhibit greater functional connectivity with SSp-bfd (Fig. 1*B*). This regional dissociation was confirmed in a linear mixed-effects model which tested for an interaction between seed-target pairs [*F* (1,56) = 12.567, *P* < 0.001]. Planned comparison pairwise *t* tests revealed significantly stronger functional connectivity between VIS targets and VISp compared with SSp-bfd [*t*(12) = 3.331, *P* = 0.006], and significantly stronger functional connectivity between SSp targets and SSp-bfd compared with VISp [*t*(16) = 2.365, *P* = 0.031].

The dissociation between seed regions and their respective visual and somatosensory targets is also evident in seedmaps of individual mice rather than group-average seedmaps (Fig. 1*C*). Whereas the group-average analysis examined RSFC between seeds and each of the target regions individually, this second analysis examined RSFC between seed regions and the entire set of target regions (VISxx or SSp-xx). To test for this circuit-level specificity, the mean functional connectivity for each seed-target grouping was calculated for each individual mouse and compared in a 2 × 2 repeated-measures ANOVA that tested for an interaction in correlation strength between seed-target pairs (VISp to VISxx, VISp to SSp-xx, SSp-bfd to VISxx, and SSp-bfd to SSp-xx). This analysis revealed a significant interaction between seeds and targets [*F*(1,30) = 361.84, *P* < 0.001; Fig. 1*C*]. Planned comparison paired-samples *t* tests confirmed that across subjects, the VIS target region set is more strongly correlated with VISp compared with SSp-bfd [*t*(30) = 15.234, *P* < 0.001], and the SSp target region set is more strongly correlated with SSp-bfd compared with VISp [*t*(30) = 15.954, *P* < 0.001]. These analyses were repeated with seeds from the opposite hemisphere, yielding similar results (*SI Appendix*, Fig. S1).

Having established that awake resting-state signals contain functionally meaningful information among two known sensory circuits in mice (see *SI Appendix*, Fig. S2 for additional comparisons), we next turn to the construction and characterization of large-scale functional brain networks in mice.

### Mouse RSFC Networks Have a Modular Architecture That Is Less Segregated in Older Age Animals.

**Fig. 2.**
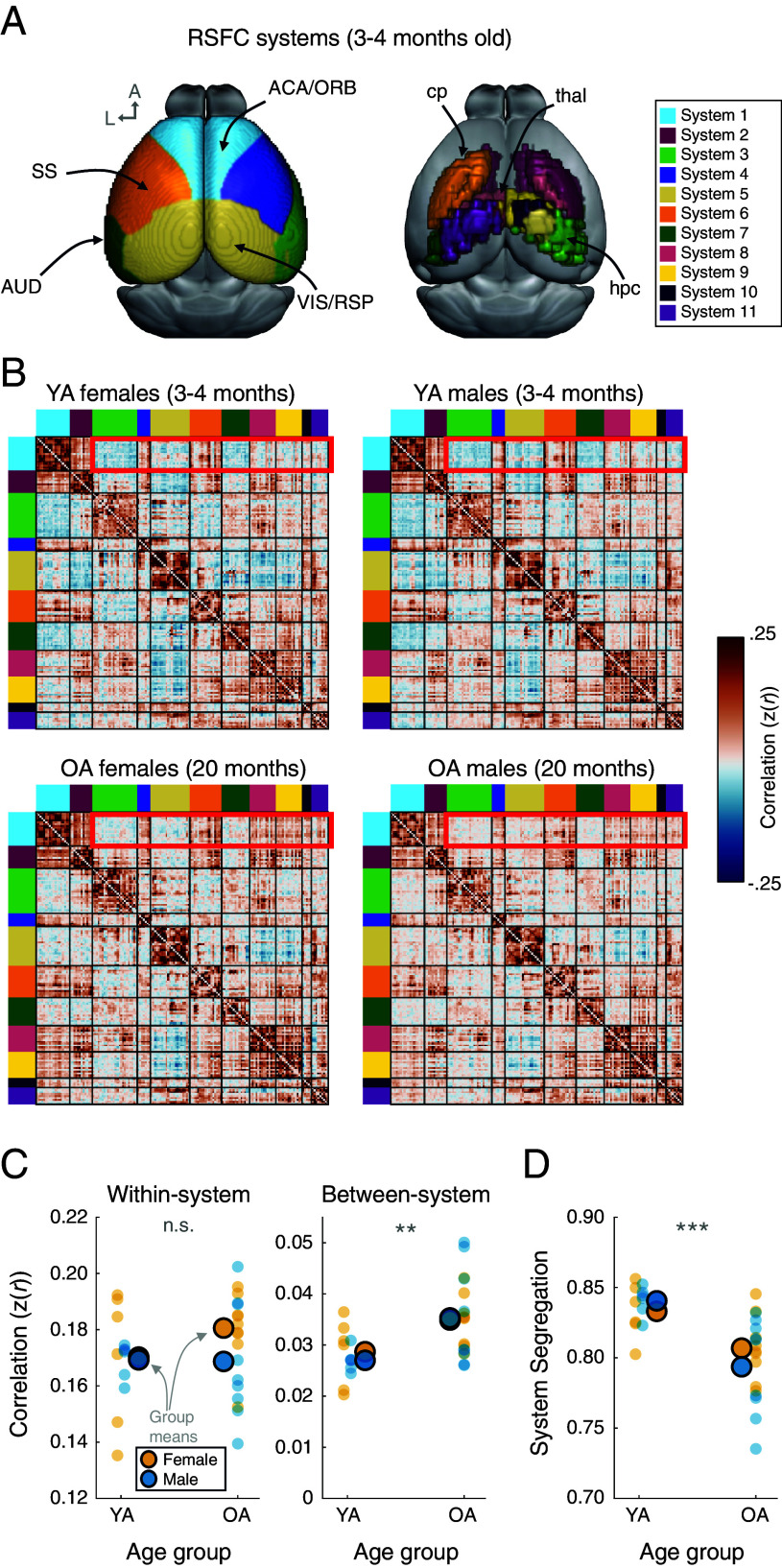
Mice exhibit modular resting-state functional network organization that is less segregated in older animals. (*A*) Functional systems of YA (3 to 4 mo old) mice were defined during resting wakefulness and mapped to brain regions from the CCFv3 atlas. These systems include numerous bilateral and/or symmetric mappings, and several correspond to established groups of functionally related regions (e.g., visual + retrosplenial; somatosensory; motor; auditory; see also *SI Appendix*, Figs. S4*A* and S6). *Left*: system labels for cortical parcels. *Right*: system labels for subcortical parcels. (*B*) Mean RSFC matrices for YA and OA mice, separated by sex and organized by the system labels in (*A*). YA male and female RSFC network matrices depict a modular organization defined by stronger within-system correlations and weaker between-system correlations. While OA male and female mice retain a modular organization, there are visibly stronger between-system correlations compared with YA mice (e.g., compare edges outlined in red). (*C*). Within-system RSFC is relatively consistent across age groups (but see *SI Appendix*, Fig. S7 for notable distinctions among certain brain systems), while between-system RSFC is stronger in OA mice. These patterns are consistent across male and female mice. (*D*) YA mice exhibit more modular network architectures than OA mice as measured by system segregation, reflecting greater differentiation of the brain systems in YA animals. The age-related differences in system segregation are consistent for males and females. SS: somatosensory cortex; AUD: auditory cortex; ACA: anterior cingulate cortex; ORB: orbital cortex; VIS: visual cortex; RSP: retrosplenial cortex; hpc: hippocampus; thal: thalamus; cp: caudateputamen. **P < 0.01; ***P < 0.001.

In humans, large-scale resting-state brain networks exhibit a modular organization wherein nodes are clustered into functionally related systems. Nodes in the same system are highly correlated with each other, and less correlated with nodes in other brain systems. Using 156 nodes defined based on a refined set of CCFv3 parcels, graph theoretic community detection was performed on a group-averaged YA (3 to 4 mo old) mouse resting-state brain network using methods established in human network neuroscience research [(53, 54); *SI Appendix*, *SI Methods—Mouse Brain Network Construction*]. This analysis revealed a robust community structure (Fig. 2*A* and *SI Appendix*, Figs. S3 and S6) which exhibits partial alignment with previously described functionally- and structurally-defined mouse brain systems [*SI Appendix*, Figs. S4 and S5; (41, 55)] while also capturing a more modular network structure than previous system labels (*SI Appendix*, Figs. S4*D* and S5*D*).

To investigate differences in network organization in relation to age and sex of mice, the RSFC network organization of 3 to 4 mo old mice (n = 12) was compared to a group of 20 mo old mice (n = 18). Fig. 2*B* depicts group-averaged matrices for each of these age groups, sorted by the functional systems depicted in Fig. 2*A* and separated by sex of each animal. From these matrices, the characteristic features of community organization can be appreciated for each group of mice. RSFCs are stronger among nodes (regions) within a community (functional system) along the diagonal (within-system relationships) and weaker/sparser among regions in different systems (off-diagonal; between-system relationships). Also evident is that compared to younger mice, older age mice exhibit stronger between-system relationships. To formally evaluate differences in within- and between-system correlations, 2 × 2 ANOVAs were conducted to test for main effects of age, sex, and their interaction on mean within- and between-system RSFC (Fig. 2*C*). Within-system functional connectivity does not show any significant differences between groups [age: F(1,26) = 0.401, *P* = 0.532; sex: F(1,26) = 1.078, *P* = 0.301; age-by-sex interaction: F(1,26) = 0.688, *P* = 0.414], while between-system functional connectivity is significantly stronger in older than in younger mice [F(1,26) = 8.609, *P* = 0.007]. Between-system functional connectivity does not differ between sexes [F(1,26) = 0.037, *P* = 0.849], nor is there an age-by-sex interaction [F(1,26) = 0.217, *P* = 0.646]. The age-related differences in mouse resting-state correlations indicate that there may exist alterations in large-scale architectures over adulthood, similar to those observed in aging human individuals (7). This hypothesis was formally tested by measuring the system segregation of individual mice. System segregation quantifies the strength of correlations within functional systems relative to those between systems, thus capturing the extent to which systems are functionally separable from each other (for review see ref. 12). As such, the measure reflects the degree of functional differentiation between distinct systems. A 2 × 2 ANOVA comparing system segregation of these four groups (Fig. 2*D*) revealed a significant effect of age wherein older mice have lower system segregation than younger mice [F(1,26) = 14.229, *P* < 0.001], but no effect of sex [F(1,26) = 0.139, *P* = 0.712] or age-by-sex interaction [F(1,26) = 1.243, *P* = 0.275].

To examine how relationships within and between specific systems and regions contribute to age-related differences, 2-sample *t* tests were conducted for each edge (i.e., node-to-node correlation value) between YA and older adult (OA) mice from this sample. These comparisons are visualized in *SI Appendix*, Fig. S8, from which it can be appreciated that age-related differences are largely bound by functional systems and their sets of relationships, and that multiple midline systems exhibit age-related declines in within-system functional connectivity in addition to broader increases in between-system functional connectivity across the network.

### Cross-Species Comparison of Brain Network Alterations across Aging Mice and Humans.

**Fig. 3.**
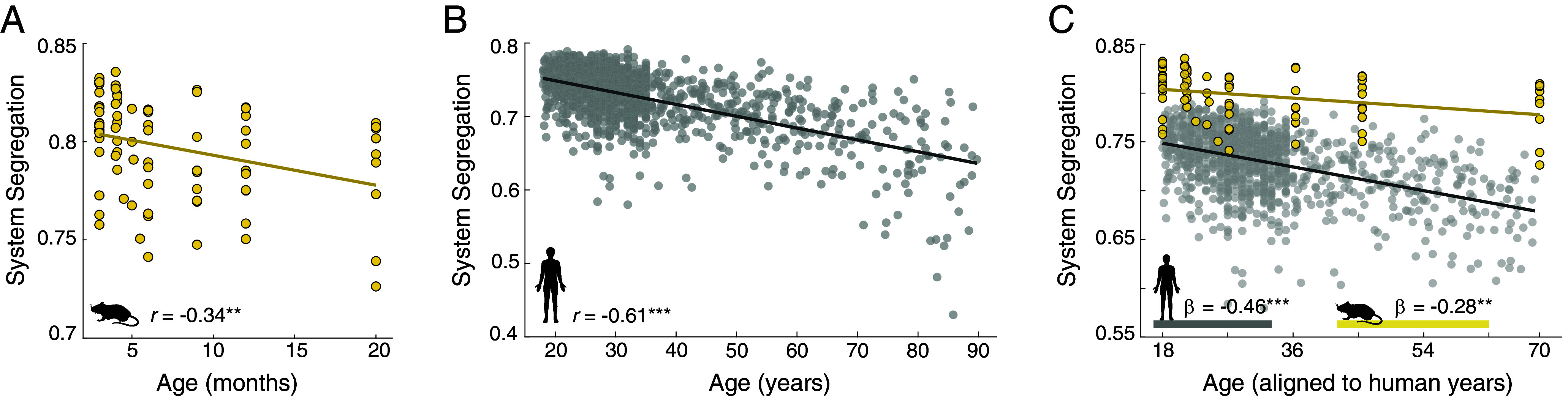
Age-related declines of system segregation in mouse and human individuals. Increasing age is associated with decreasing resting-state system segregation in both (*A*) mouse and (*B*) human individuals. Each dot is an individual. (*C*) Direct comparison of mouse and human system segregation trajectories over a broad range of adulthood reveals that mice exhibit higher functional segregation than humans and have a less rapid rate of decline than humans. β values listed in the figure panel come from the cross-species model. Here, 3 mo old mice are considered to be equivalent to 18 y old humans and 20 mo old mice are considered to be equivalent to 70 y old humans, based on previously reported developmental and aging mappings across species [Flurkey et al. (48)]. Refer to *SI Appendix*, Fig. S15 for alternate age mappings. **P < 0.01; ***P < 0.001.

In humans, system segregation exhibits continuous declines over the range of adulthood. An expanded set of mouse data, comprising intermediate ages between the 3 and 20 mo ages initially tested and including a combination of both cross-sectional and longitudinal data, was included to evaluate the trajectory of age-related differences and changes in system segregation across a wide range of mouse adulthood. Fig. 3*A* depicts the resting-state system segregation of each mouse spanning from 3 to 20 mo. A linear mixed-effects model including age and sex, as well as longitudinal information about repeatedly scanned animals, confirmed a main effect of age on system segregation [F(1,82) = 9.307, *P* = 0.003], such that system segregation declines over the course of mouse adulthood. There is no effect of sex on system segregation [F(1,82) = 0.022, *P* = 0.882]. Critically, the age-related decline of system segregation remains consistent across a range of analytic decisions involving choice of node sets, network densities, and preprocessing steps, supporting the robustness of the observations to various technical and methodological considerations (*SI Appendix*, Figs. S9–S13).

Focusing in on a subset of mice for which longitudinal data was acquired beginning at 6 mo (n = 10), with additional scans at 9 mo (n = 10) and 12 mo (n = 6), we calculated changes in system segregation across time. While this age range corresponds to a limited window of adulthood, a linear mixed-effects model revealed that system segregation declines as a function of increasing age within individual mice [F(1,23) = 10.613, *P* = 0.003; *SI Appendix*, Fig. S14].

Aging mice exhibit large-scale resting-state network alterations that appear similar to those previously reported in human participants. To evaluate this correspondence directly, resting-state brain system segregation was calculated in human individuals obtained from the Human Connectome Project Young Adult (HCP-YA) and a subset of its developmental (HCP-D) and aging counterparts (HCP-A), fusing the data collected across efforts to include participants over a broad range of adulthood (age range: 18 to 90 y). Consistent with past findings but now using more robust estimates of brain network organization enabled by advances in data acquisition (56–58) and processing (59), age-related decline in system segregation is evident across human adulthood (*r* = −0.608, *P* < 0.001, Fig. 3*B*).

It is clear that both adult mice (Figs. 2*D* and 3*A*) and humans (Fig. 3*B*) exhibit age-accompanied declines in the segregation of their large-scale resting-state brain networks. Utilizing a common analytical framework for examining brain network organization enables cross-species comparison of age-related large-scale brain network trajectories; this level of comparison is agnostic to the presence or absence of homologous brain areas or systems across species but rather is focused on features and age-related alterations in measures of global brain network topology.

Age-accompanied network alterations were compared across species based on proposed correspondence of developmental timelines [i.e., 3 to 20 mo old mice vs. 18 to 70 y old humans; (47, 48); Fig. 3*C*]. A linear mixed-effects model tested the main effects of species, age, and their interaction on system segregation, while controlling for sex. There is a main effect of age wherein older age is associated with lower system segregation, irrespective of species [*F*(1,1187) = 6.053, *P* = 0.014]. In addition, there is a main effect of species: human individuals exhibit lower system segregation, irrespective of age [*F*(1,1187) = 27.959, *P* < 0.001]. These main effects are accompanied by a species-by-age interaction [*F*(1,1187) = 16.888, *P* < 0.001] wherein mice exhibit a less steep pattern of brain network aging relative to humans (mouse *β*: −0.280, human *β*: −0.457; *β* values come from the cross-species model). The correspondence between human and mouse age is admittedly uncertain (60) and by definition influences the cross-species age comparisons. We explored alternate age mappings across species; main effects of age and species and their interaction are robust to alternative age mappings (*SI Appendix*, Fig. S15) and signal processing considerations (*SI Appendix*, Fig. S16).

### Cross-Species Differences in System Segregation Are Related to Differences in Long-Range Functional Connectivity between Distributed Brain Systems.

**Fig. 4.**
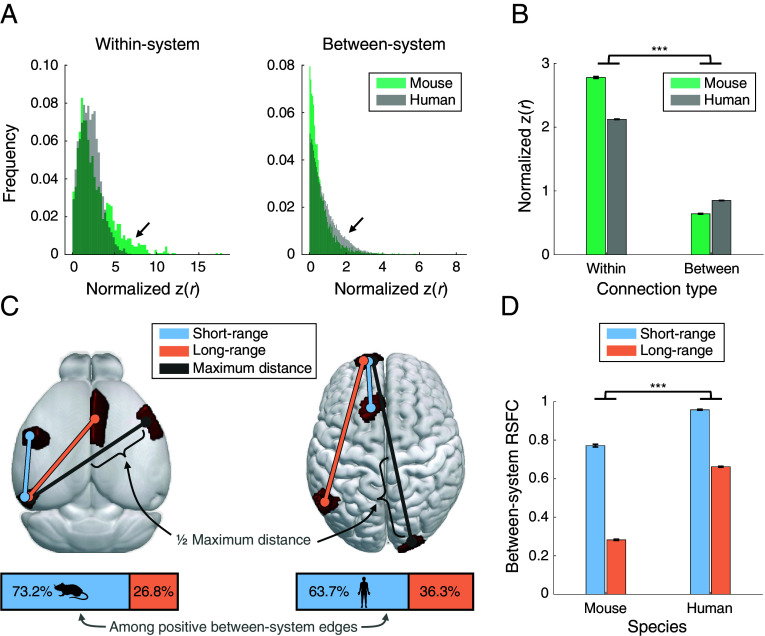
Cross-species differences in system segregation reflect reduced long-range integration of RSFC systems in mice. (*A*) YA mice show stronger within-system RSFC than humans, while YA humans exhibit stronger between-system RSFC than mice (see *SI Appendix*, Fig. S17 for analogous comparisons in older age individuals). Arrows highlight these differences between species. (*B*) Although within-system RSFC exceeds between-system RSFC in both species, the contrast is significantly greater in mice, leading to higher system segregation. Error bars reflect SE. (*C*) Between-system edges were categorized according to the physical distance between each pair of regions (short-range vs. long-range, based on whether the distance was shorter or longer than half the maximum distance of region-to-region relationships measured for that species). Horizontal stacked bars below depict proportions of functional connection types: mice have a lower proportion of long-range between-system relationships compared to humans, indicating reduced capacity for long-distance integration across brain systems. (*D*) The distance-based distinction is also evident from comparisons of RSFC strength. Bar plots depict mean RSFC strength of short-range vs. long-range between-system relationships: among between-system edges, mice show a greater difference in functional connectivity strength as a function of physical distance, compared to humans. Error bars reflect SE. ***P < 0.001.

Resting-state system segregation is higher in mice than in humans. This difference may reflect stronger within-system functional connectivity, weaker between-system functional connectivity, or both. To evaluate these possibilities, we directly compared RSFC patterns across species using YA RSFC matrices (*SI Appendix*, *SI Methods—Brain Network Analysis*). A 2 × 2 ANOVA assessed RSFC strength as a function of species and connection type (within-system vs. between-system; Fig. 4 *A* and *B*), revealing significant main effects of species [F(1,45772) = 197.66, *P* < 0.001] and connection type [F(1,45772) = 11476.34, *P* < 0.001], as well as a species-by-connection type interaction [F(1,45772) = 736.86, *P* < 0.001]. As expected, within-system RSFC exceeds between-system RSFC in both species. However, mice show stronger within-system RSFC than humans, while humans exhibit stronger between-system RSFC than mice. Differences in within-system vs. between-system RSFC are thus more pronounced in mice, contributing to their higher overall system segregation (Fig. 3*C*). Notably, this pattern is also evident in older age groups (*SI Appendix*, Fig. S17).

Previous anatomical studies have reported that mice possess fewer long-range cortico-cortical structural connections compared with primates (61), which often link distributed brain systems (62). Based on this observation, we hypothesized that cross-species differences in between-system RSFC would be especially pronounced for long-range relationships. We classified between-system RSFC edges as short- or long-range based on Euclidean distance between regions, within each species. A 2 × 2 ANOVA on RSFC strength across these distance-defined RSFC categories (Fig. 4 *C* and *D*) revealed main effects of species [F(1,40020) = 556.14, *P* < 0.001] and functional connection distance [F(1,40020) = 1071.42, *P* < 0.001), along with a significant species-by-distance interaction [F(1,40020) = 65.09, *P* < 0.001). Mice exhibit weaker RSFC among long-range between-system relationships relative to short-range relationships, in contrast to humans, who show less distinct distributions of long- vs. short-range between-system RSFC. These patterns are again evident in older age (*SI Appendix*, Fig. S17), altogether reflecting greater functional integration of distributed large-scale systems in humans compared to mice, across adulthood.

## Discussion

The mouse resting-state brain network is organized into a set of segregated subnetworks, or brain systems. These systems are composed of functionally correlated brain areas, and the organization only partially overlaps with subnetworks previously defined by axonal connectivity, indicating distinctions in the organization of large-scale functional networks compared to networks defined from structural features. This large-scale functional brain network organization varies systematically with age over the mouse adult lifespan. A combination of cross-sectional and longitudinal comparisons demonstrates that increasing adult age is associated with declining system segregation—a measure summarizing the modular organization of the brain—mirroring the functional dedifferentiation of resting-state brain network organization observed in aging humans. Despite this similarity, mouse brain networks are more segregated than human brain networks irrespective of age, a property which can be partially attributed to weaker long-range between-system relationships among mice. In addition, mice exhibit less rapid age-related declines in system segregation compared with humans. Our description of aging-related differences in the functional brain network of mice serves as a translational bridge across species and spatial scales of enquiry. We discuss the implications of these observations toward characterizing functional specialization in the mouse brain network and cross-species models of age-related brain decline.

The brain’s modular organization supports the functional specialization of its network components (2). In human individuals, large-scale resting-state networks are organized into modules that correspond to known functional systems (63, 64), and the segregation of these systems supports their distinct processing operations (12, 65). Modular resting-state brain networks have been described in the mouse, providing evidence for multiple RSFC systems, including a medial system comprising retrosplenial and cingulate areas and a lateral system primarily encompassing sensory-motor regions (for review see ref. 35). Parts of this organization align with descriptions of anatomical subnetworks (55). These descriptions have been made primarily in younger adult mice [cf. (66)]. Descriptions of resting-state network architecture have also typically been made with mice under anesthesia (38, 66), which has been shown to exhibit differences from awake protocols (46). Although correspondence between anatomical and functional connectivity is observed under anesthesia (36, 43) and in awake mice (34), this correspondence is reduced in the awake state (46), indicating that brain state systematically alters functional network organization. Similar state-dependent effects have been reported in nonhuman primates (67), and anesthesia-related alterations of resting-state networks have been reported in humans (68, 69). Since most human resting-state data are acquired during wakefulness (e.g., refs. 6 and 70), the use of anesthesia in mouse resting-state fMRI represents a potential confound that limits cross-species comparison. The awake imaging protocol and adult lifespan sampling employed here provided an opportunity to directly evaluate and compare resting-state brain network organization and its age-related alterations across mice and humans.

Mouse resting-state networks are more segregated than human networks across all examined ages, a pattern that could reflect greater functional specialization, reduced intersystem integration, or both. This difference is driven in part by lower between-system correlations in mice compared with humans (Fig. 4*A*), resulting in greater functional separation among systems. In humans, greater integration across systems—particularly those supporting control operations—has been attributed to the demands of dynamically coordinating and processing multimodal inputs and outputs across the network (63, 71). In mice, a disproportionate fraction of the cortex is devoted to sensory processing [particularly somatomotor regions (72, 73)], relative to integrative functions that are characteristic of the primate association cortex (74–76). Accordingly, higher system segregation in mice may reflect a lesser need and/or capacity for higher-order integrative processing across the network, or species-specific selective pressures that have optimized alternative processing demands (77). Together, these species-level differences in intrinsic network organization may shape the magnitude and pattern of age-related changes in segregation and integration within each species.

In contrast to the present functional connectome observations, an analysis of structural connectivity revealed the opposite pattern: structural brain networks are more segregated in primates than in rodents (78). This pattern has been interpreted as reflecting the increasing cost of long-range connections with greater brain volume, which constrains neural wiring toward more local connectivity and, consequently, a more modular network organization (78). Interestingly, humans appear to violate this constraint by possessing association systems that include highly connected, spatially distant cortical areas despite the substantial cost of maintaining long-range connections (for review see ref. 62). Consistent with this possibility, distance-dependent analyses indicate that although functional connectivity is generally stronger between nearby regions compared with distant regions for both species, this effect is more pronounced in mice than in humans (Fig. 4*D*). Altogether, these findings point to a common tradeoff underlying distributed association systems in humans and the divergence between structural and functional modularity across species: while wiring cost constraints strongly shape structural organization across mammals (79, 80), the benefits of higher-order integrative processing in humans drive stronger long-range functional relationships, enabling greater integration across distributed systems and, consequently, lower functional system segregation (81). Extending this work to nonhuman primates will be an important next step in clarifying how long-range functional relationships contribute to cross-species differences in brain network organization and how wiring constraints, integrative demands, and ecological pressures jointly shape large-scale brain network organization across species.

Declining system segregation is a hallmark of human aging; it is associated with worse cognitive ability in healthy adults (7, 9, 82–85), greater cognitive dysfunction in patient populations (17, 18), and is prognostic of dementia in aging individuals (16). Age-related reductions in system segregation are also accompanied by a dedifferentiation of task-related areal activity patterns (13), indicating that the functional connectivity alterations are related to information processing during goal-directed tasks.

In mice, progressive reductions in system segregation were evident beginning at 3 mo of age and continued across subsequent phases of adulthood. An intriguing possibility is that this large-scale dedifferentiation is mechanistically related to analogous cellular-level processes. Aging in mice is accompanied by reduced selectivity of sensory neurons across multiple sensory modalities (86–88), a pattern also observed in nonhuman primates (89, 90). The trajectory of large-scale brain network decline in mice also parallels age-related reductions in the differentiation of excitatory synapses across cortical and subcortical regions, which begin around 3 mo of age (91). These findings point to a general process of functional dedifferentiation over adulthood, occurring at multiple levels of neural organization, from neurons to whole-brain networks. One possibility is that age-related decline at the cellular and network levels are causally linked, with one giving rise to the other; alternatively, both may independently arise from common upstream drivers (e.g., environmental stress or inflammation). Scale-specific manifestations may reflect emergent properties, where higher-level patterns arise from but cannot be fully reduced to lower-level processes. Bridging theories of brain aging across organizational levels remains a fundamental challenge in neuroscience (92). The present findings offer an empirical link between cellular- and network-level dedifferentiation, providing a foundation for future work aimed at connecting these levels to explain age-related changes in behavior and cognition.

Direct comparison of mouse and human system segregation trajectories revealed slower functional network aging in mice compared with humans. Mouse-human age alignments can vary depending on the behavioral phenotype (25), population measure (48), or disease pathology (93) under consideration. In keeping with this, we explored a range of age alignments to better characterize cross-species similarities and differences in trajectories of functional brain network aging. The cross-species differences were robust across age alignments (*SI Appendix*, Fig. S15). More broadly, system segregation and RSFC network organization may serve as useful neurophysiologic benchmarks for informing cross-species correspondence of developmental and aging timelines. While our current scanning protocol precludes fMRI acquisition in mice younger than 3 mo due to incomplete cranial growth which prohibits necessary procedures, an important direction for future research will be to examine the development of functional network organization across earlier life stages (see ref. 94 for related work using calcium imaging).

Given the numerous differences between cortical organization at the transcriptomic (95), cellular (61), areal (96, 97), and connectomic (61) levels of brain structure and function, in addition to fundamental cognitive differences (98), there are challenges in establishing direct homologies between specific areas, circuits, and systems across mice and humans (see ref. 99 for an overview of efforts in this direction). Indeed, the very fact that mice are not just small people necessitates some limit to cross-species mappings. While the areal- and systems-level descriptions of organization are inarguably critical domains of cross-species research, we have explicitly avoided these comparisons in the current work and instead have aimed to characterize and interpret cross-species similarities and differences at the level of large-scale network topology. While undoubtedly constrained-by and related-to lower levels of organization and function, the measurement and interpretation of topological features of network organization such as system segregation circumvents difficulties of interpretation that are present at the systems level, which require greater emphasis on areal homologies that may not always exist [(100), but see *SI Appendix*, Fig. S8 for comparisons along these lines, focused on areas implicated in the human default network]. Rather, system segregation describes the extent of modular organization, only assuming that this feature is conducive to overall brain function and that changes in this measure of network organization are reflective of alterations in the functional specialization of its components, regardless of the exact processing roles of the components themselves. This feature makes the measure suitable for comparison across differing species and even types of networks (e.g., biological, technological, social). We and others have also argued that this level of description may be more intimately linked to general measures of complex behavioral phenotypes (12, 77) and that it relates to differences in environmental exposures and cognitive dysfunction (16) in humans. Establishing a mouse model of age-related changes in large-scale brain network organization catalyzes experimental testing of these hypotheses given the feasibility of direct experimental control and manipulation of cellular, genetic, and environmental factors across the relatively short lifespan of mice. This supports the use of system segregation as a biomarker of overall brain function across the lifespan in the mouse, complementing other health biomarkers focused on metabolism (101, 102), cellular function (103), and brain structure (104), and possibly constituting a general hallmark of brain aging (105).

Importantly, our approach has implications for research programs targeting pathological as well as healthy aging. Despite a rich literature linking AD pathology to functional connectivity in humans (17, 18, 106) and mice (31–33), many AD-targeting drugs that show promise in animals fail in human clinical trials. This translational gap underscores the need to characterize and directly compare brain function across species at both cellular and large-scale network levels. Measures of resting-state network organization provide a principled framework for enabling such cross-species comparisons.

Finally, it is important to acknowledge limitations that constrain the interpretability of the results reported here. Most notably, differences between mouse and human data—including fMRI sequences, voxel size, data amount, and environment—have all been linked to differences in RSFC network properties and are unavoidable in the present dataset, despite our efforts to apply comparable preprocessing and analytic pipelines across species. Importantly, however, the convergence in age-related trajectories of system segregation observed in mice and humans suggests that this measure captures a highly robust phenotype of aging brain function.

## Conclusion

Age-related decline in large-scale brain network organization is a key feature of cognitive aging and neurodegenerative disease in humans, yet its mechanistic origins and modulators remain poorly understood due to inherent constraints in human research. Here, we identify an analogous pattern of resting-state functional network decline in awake mice, establishing a cross-species signature of brain aging that is both experimentally tractable and translationally relevant. This mouse model of large-scale network aging enables targeted manipulations at molecular, cellular, and circuit levels, providing a powerful framework to link neural systems to behavior. Having identified a shared network phenotype across species, future work should focus on delineating the factors that confer vulnerability or resilience to age-related network decline. Such efforts will yield mechanistic insight into the causes and consequences of functional brain aging and promote productive, bidirectional exchange between human and animal models. Ultimately, coordinated cross-species investigations across multiple levels of organization will be essential for uncovering the biological principles that govern aging, cognition, and disease.

## Materials and Methods

### Mouse and Human Neuroimaging Datasets.

Detailed methods can be found in *SI Appendix, SI Methods*. Five mouse and three human neuroimaging datasets were included in this project. Mouse data were collected at Technion—Israel Institute of Technology and Columbia University (C57BL/6; total N = 82). All procedures were conducted in accordance with the ethical guidelines of the National Institutes of Health and were approved by the institutional animal care and use committee at Technion—Israel Institute of Technology or Columbia University. Human datasets include the Human Connectome Project Young Adult [HCP-YA; (107)], the Human Connectome Project Aging [HCP-A; (56)], and the Human Connectome Project Developmental [HCP-D; (58)] datasets (total N = 1,179). The scanning protocol for humans was approved by the Washington University in St. Louis’s Human Research Protection Office and all participants provided written informed consent. Only deidentified data were downloaded and used in the current study. Additional information regarding these datasets is available in *SI Appendix*, *SI Methods—Mouse Data Acquisition and Processing* and in *SI Methods—Human Data Acquisition, Processing, and Network Construction*.

### Mouse Data Acquisition and Preprocessing.

Mouse neuroimaging at the Technion site was conducted using a Bruker 9.4T scanner with a quadrature 86 mm transmit-only coil and a 20 mm loop receive-only coil. Mouse imaging at the Columbia site was conducted using a Bruker 9.4T scanner with a quadrature 86 mm receive-only surface array coil. Mice at both sites were implanted with MRI compatible headposts and acclimatized to the scanner over several sessions, after which they were scanned across multiple 33-min-long awake functional imaging sessions. Each animal had at least one structural MRI scan. Scanning protocols followed previous work in awake mice (34, 42, 108–110) and are detailed further in *SI Appendix*, *SI Methods—Mouse Data Acquisition and Processing*.

Where possible, mouse fMRI preprocessing steps were aligned with those performed in humans, in order to support cross-species interpretability. Mouse fMRI data preprocessing included NOise Reduction with DIstribution Corrected [NORDIC; (111–113)], standard fMRI preprocessing using Rodent Automated Bold Improvement of EPI Sequences [RABIES; (114)], and resting-state fMRI-specific preprocessing using RABIES (confound correction step). Briefly, preprocessing included censoring of high-motion frames, regression of motion parameters and tissue signals (including the global signal, but also see *SI Appendix*, Fig. S16 for analysis without this processing step), bandpass filtering, and spatial smoothing. Anatomical images were corrected for inhomogeneity and registered to the Dorr-Steadman-Ulman-Richards-Qiu-Egan atlas (115–118). Functional images from the Columbia site were all resliced to 150 × 150 × 450 µm^3^ to match data from the Technion site. Additional details can be found in *SI Appendix*, *SI Methods*—*Mouse Data Acquisition and Processing*.

### Human Data Acquisition and Preprocessing.

Human neuroimaging was conducted using 3T scanners with 32-channel head coils (HCP-YA: customized Siemens 3T Skyra; HCP-D/HCP-A: Siemens 3T Prisma). Each participant completed two scanning sessions on separate days, which included awake resting-state fMRI data collection (HCP-YA: four total scans of 14.4 min; HCP-D/HCP-A: four total scans of 6.5 min). Structural scans were also collected for each participant. Detailed scan parameters can be found in *SI Appendix*, *SI Methods—Human Data Acquisition, Processing, and Network Construction*.

Human MRI images were processed using HCP Pipeline processing. MRI preprocessing employed the HCP Pipeline’s fMRI Volume processing, in-house resting-state fMRI-specific preprocessing steps, and HCP Pipeline’s fMRI Surface processing. Resting-state preprocessing was applied to reduce spurious variance unlikely to reflect neuronal activity in RSFC data (70). As with mice, this included censoring of high-motion frames, regression of motion parameters and tissue signals (including the global signal, but also see *SI Appendix*, Fig. S16 for analysis without this processing step), bandpass filtration, and spatial smoothing. Additional details can be found in *SI Appendix*, *SI Methods—Human Data Acquisition, Processing, and Network Construction*.

### Mouse and Human Network Construction and Analysis.

Mouse network nodes were defined using the Allen Institute Mouse Brain Common Coordinate Framework [CCFv3; (51)]. Adjustments to the CCFv3 parcel definitions were conducted as detailed in *SI Appendix*, *SI Methods—Mouse Brain Network Construction*, to account for known functional distinctions within several nodes. Human network nodes were defined using the Schaefer 400 parcellation (119).

Correlation matrices were constructed for each mouse and human individual by averaging blood-oxygen-level-dependent (BOLD) timeseries within each network node, yielding a n × t timeseries for each individual, where n is the number of nodes and t is the number of frames. Number of frames were equated across individuals within each species (59). These timeseries were used to form a n × n matrix of Fisher’s z-transformed Pearson’s correlation values between each pair of nodes, and the diagonal of the matrix was set to 0.

To account for differences in the protocol and head coil used across acquisition sites (in mice) and studies (in humans), Correcting Covariance Batch Effects [CovBat, (120)] was employed to harmonize correlation matrices prior to statistical analysis.

Community detection in mice was performed using the Infomap algorithm (53) across a range of edge densities (0.5 to 20%, increments of 0.5%). Outputs from Infomap were adjusted to yield final functional system labels across a range of densities, following previous approaches used in human research (54). Results reported in the main text are based on communities detected at 7% edge density; results using other densities are presented in *SI Appendix*, Fig. S9. In all primary analyses, communities were derived from the group-average matrix of YA (3 to 4 mo old) mice (see *SI Appendix*, Fig. S7 for analyses based on age-group specific communities). Additional details are provided in *SI Appendix*, *SI Methods—Mouse Brain Network Construction*. Human functional systems were defined using the “Kong2022 17 network” community label (121), which is based on HCP-YA data (i.e., functional systems were defined in-sample for both mice and humans).

### Brain Network Analysis.

For each species, system segregation was calculated on the weighted matrices of individuals (mice and humans), with negative values set to zero. System segregation quantifies the extent to which an individual’s brain network is clustered into distinct systems, according to the following equation:

$$\mathrm{system}\;\mathrm{segregation}=\frac{\frac{\sum_{w}^{W}Z_{w}}{W}-\frac{\sum_{b}^{B}Z_{b}}{B}}{\frac{\sum_{w}^{W}Z_{w}}{W}},$$

where Z_w_ represents Fisher’s z-transformed correlation values between nodes in the same system, Z_b_ represents correlation values between nodes belonging to different systems, W represents the total number of within-system edges in the network, and B is the total number of between-systems edges in the network. Thus, system segregation measures the relative magnitude of within-system edges compared with between-system edges (7). As system segregation is calculated on a single correlation matrix, values are computed for each individual mouse and human. Degrees of freedom reported in comparisons of system segregation thus reflect individual subjects.

Linear mixed-effects models were applied to estimate slopes of system segregation differences (cross-sectional) and changes (longitudinal) across adulthood in mice, as well as to compare trajectories of system segregation between mice and humans. Additional details regarding statistical models and analyses are provided in *SI Appendix*, *SI Methods—Brain Network Analysis*.

## Supplementary Material

Appendix 01 (PDF)

## Data Availability

The human data used in this study are publicly available. The HCP-D dataset can be downloaded from: https://www.humanconnectome.org/study/hcp-lifespan-development/data-releases (58), the HCP-YA dataset can be downloaded from: https://www.humanconnectome.org/study/hcp-young-adult/data-releases (107), and the HCP-A dataset can be downloaded from https://www.humanconnectome.org/study/hcp-lifespan-aging/data-releases (56). Mouse data used in the study are publicly available in BIDS format at https://openneuro.org/datasets/ds007100 (122). Functional system labels and adjusted ROIs used in the primary and supplementary analyses, respectively, are available at https://gitlab.com/wiglab/mouse-community-nodes (123). Code for system segregation calculation is available at: https://gitlab.com/wiglab/system-segregation-and-graph-tools (124). Code for NORDIC used in mouse preprocessing is available at: https://github.com/SteenMoeller/NORDIC_Raw (112). Code for RABIES used in mouse preprocessing is available at: https://github.com/CoBrALab/RABIES (114). Code for the HCP-fMRI preprocessing pipeline is available at: https://github.com/Washington-University/HCPpipelines (125).
