# Supplementary material for: Correspondence of large-scale functional brain network decline across aging mice and humans
Source: Proc Natl Acad Sci U S A. 2026 Mar 27;123(13):e2527522123. doi: 10.1073/pnas.2527522123 (PMC13037878; doi:10.1073/pnas.2527522123)
Supplement: Supplementary file 1 — Appendix 01 (PDF) [file pnas.2527522123.sapp.pdf]

PNAS

Supplementary Information (SI) for:

# Correspondence of large-scale functional brain network decline across aging mice and humans

Ezra Winter-Nelson[1,2], Eyal Bergmann[3,4], Micaela Y. Chan[1], Gabriella Vill[4], Liang Han[1], Ziwei Zhang[1], Alexandra Kavushansky[3], Irit Dolgopyat[3], Jad Asleh[3], Jennifer D. Whitesell[5], Itamar Kahn[4], & Gagan S. Wig[1,2,6]

[1]Center for Vital Longevity, The University of Texas at Dallas, Dallas, TX, USA
[2]Department of Psychology, School of Behavioral and Brain Sciences, The University of Texas at Dallas, Dallas, TX, USA
[3]Rappaport Faculty of Medicine, Technion – Israel Institute of Technology, Israel
[4]Zuckerman Mind Brain Behavior Institute and Department of Neuroscience, Columbia University, New York, NY, USA
[5]Allen Institute for Brain Science, Seattle, Washington, USA
[6]Department of Psychiatry, The University of Texas Southwestern Medical Center, Dallas, TX, USA

Corresponding author: Gagan S. Wig, Ph.D.
Email: gwig@utdallas.edu

This PDF file includes:

SI Methods
SI Results (Figures S1 to S17, Table S1)
SI References

1. SUPPLEMENTARY METHODS ***3***

DATASETS **3**

MOUSE DATA ACQUISISTION AND PROCESSING **3**

MOUSE BRAIN NETWORK CONSTRUCTION **4**

HUMAN DATA ACQUISITION, PROCESSING, AND NETWORK CONSTRUCTION **6**

BRAIN NETWORK ANALYSIS **8**

2. SUPPLEMENTARY RESULTS ***11***

2.1 – *Specificity of mouse RSFC patterns using left hemispheric seeds.* **11**

2.2 – *Regions exhibit strong homotopic resting-state functional correlations.* **13**

2.3 – *Nodes and communities included in system segregation calculation across edge densities* **14**

2.4 – *Comparison of resting-state systems (community organization) with structurally-defined system labels.* **15**

2.5 – *Comparison of community organization with alternate functionally-defined community labels.* **17**

2.6 – *Modular functional organization of the mouse brain.* **19**

2.7 – *Age-related declines in RSFC system segregation using age-group specific system labels.* **20**

2.8 – *Age-related RSFC network alterations are evident within and between several systems.* **21**

2.9 – *Age-related declines in system segregation are evident using system labels (communities) defined across a range of edge density values.* **22**

2.10 – *Age-related declines in system segregation are evident when global signal regression (GSR) is not included in resting-state preprocessing.* **23**

2.11 – *Age-related declines in system segregation are evident when using all available clean BOLD data volumes.* **24**

2.12 – *Age-related declines in system segregation are evident when using network nodes defined from a refined areal parcellation.* **25**

2.13 – *Age-related system declines in system segregation are evident when using an alternate community detection algorithm.* **27**

2.14 – *Longitudinal declines of resting-state system segregation within individual mice during early middle age.* **29**

2.15 – *Effects of alternate mouse-human age alignments on cross-species system segregation comparisons.* **30**

2.16 – *Comparison of mouse and human system segregation trajectories in the absence of global signal regression (GSR)* **31**

2.17 – *Cross-species differences in system segregation reflect reduced long-range integration of RSFC systems in older age mice.* **33**

3. SUPPLEMENTARY REFERENCES ***36***

1. SUPPLEMENTARY METHODS

DATASETS

Five mouse and three human neuroimaging datasets were included in this project. Mouse data was collected at Technion – Israel Institute of Technology and Columbia University, described below. The five mouse studies were acquired over the course of multiple years, but handling, acquisition, and preprocessing protocols were nearly identical for all mice (minor acquisition details differed between the two imaging sites, detailed below). Human datasets include the Human Connectome Project Young Adult (HCP-YA; (1)), the Human Connectome Project Aging (HCP-A; (2)), and the Human Connectome Project Developmental (HCP-D; (3)) dataset. These datasets were combined and harmonized (detailed below) in order to characterize human lifespan trajectories of brain network alterations and to match the range of ages examined in the mouse datasets.

MOUSE DATA ACQUISISTION AND PROCESSING

Mice, surgical procedures, acclimatization

All procedures were conducted in accordance with the ethical guidelines of the National Institutes of Health and were approved by the institutional animal care and use committee (IACUC) at Technion – Israel Institute of Technology or Columbia University. A total of 52 mice were scanned at the Technion Site. Scanning was conducted on C57BL/6 mice of different ages: 19 mice aged 3 months (19 males), 14 mice aged 4-6 months (6 males, 8 females), 8 mice aged 12 months (8 males) In addition, a group of mice (all males) were scanned longitudinally at age 6-7 months (n=11), age 9-10 months (n=10), and age 12-13 months (n=5). In the longitudinal cohort, one animal was lost to attrition due to infection of the headpost implant at the second timepoint, and an additional 5 animals were lost for the same reason at the third timepoint. Scanning conducted at the Columbia University site included a group of young adult mice (3-4 months; n=12; 6 males, 6 females) and a group of older adult mice (20 months; n = 18; 9 males, 9 females).

Surgical, handling, and acclimatization protocols were identical at both scanning sites. Mice were implanted with MRI-compatible head-posts (4) and housed in a reversed 12-hour light/dark cycle. Following a period of recovery (at least 3 days), mice were acclimatized to awake functional MRI (fMRI) during passive wakefulness (resting-state) over 4 separate sessions (lasting 2, 5, 10 and then 25 minutes). Following acclimation, neuroimaging involved multiple 33-minute-long awake functional imaging sessions (mean number of sessions: 6.2 ± 2.5) over a range of days (3 to 31 days). Each animal had at least one structural MRI scan. Mice underwent structural imaging prior to functional scans during each session. Mice were visually monitored during and after the scan to confirm wakefulness. Following data acquisition, mice were unmounted from the cradle and returned to their cages.

Mouse structural and functional MRI acquisition

Technion site: MRI scans were performed at 9.4 Tesla MRI (Bruker BioSpin GmbH, Ettlingen, Germany) using a quadrature 86 mm transmit-only coil and a 20 mm loop receive-only coil (Bruker); raw fMRI data were reconstructed using ParaVision 5.1 (Bruker). The scanning protocol was similar to previous awake scanning experiments conducted by members of our group and others (4–8). In each fMRI session, mice were briefly anesthetized (5% isoflurane) and mounted to a custom-made cradle (4). Mice were usually fully awake in less than a minute and had ~15 min to recover during scanner calibrations and acquisition of structural images. Structural images used the relaxation enhancement (RARE) T2-weighted sequence (TR = 1500 ms, TE = 8.5 ms, RARE-factor = 4, FA = 180°, 30 coronal slices, 150 × 150 × 450 μm^3 voxels, no interslice gap, FOV 19.2 × 19.2 mm^2, matrix size of 128 × 128). Following the structural scan, four spin-echo echo-planar imaging (SE-EPI) runs (awake resting-state fMRI) were acquired (TR = 2500 ms, TE = 18.398 ms, 200 time points, FA = 90°, 30 coronal slices, 150 × 150 × 450 μm^3 voxels, no interslice gap, FOV 14.4 × 9.6 mm^2, matrix size of 96 × 64). Several mice (n=14) were imaged at a slightly different resolution (150 × 150 × 400 μm^3 voxels or 200 × 200 × 200 μm^3 voxels); all other parameters were the same, and images were resliced to 150 × 150 × 450 μm^3.

Columbia site: MRI scans were performed using a 9.4 Tesla MRI (Bruker BioSpin GmbH, Ettlingen, Germany) using a quadrature 86mm receive-only surface array coil (Bruker); raw fMRI data were reconstructed using ParaVision 6.0.1 (Bruker). Structural images used RARE T2-weighted sequence (TR = 2300 ms, TE = 8.5 ms, RARE-factor = 4, FA = 180°, 50 coronal slices, 200 × 200 × 300 μm^3 voxels, no interslice gap, FOV 19.2 × 19.2 mm^2, matrix size of 96 × 96). Following this structural scan, four SE-EPI runs (awake resting-state fMRI) were acquired (TR = 2500 ms, TE = 13.022 ms, 200 time points, FA = 90°, 50 coronal slices, 200 × 200 × 300 μm^3 voxels, no interslice gap, FOV 14.4 × 9.6 mm^2, matrix size of 72 × 48).

Mouse structural and functional MRI preprocessing

Where possible, we implemented mouse fMRI processing steps that were comparable to those performed in humans, in order to maximize cross-species interpretability. Mouse fMRI data preprocessing included NOise Reduction with DIstribution Corrected (NORDIC; (9–11)), standard fMRI preprocessing, and resting-state specific preprocessing. NORDIC was implemented through MATLAB (https://github.com/SteenMoeller/NORDIC_Raw) to reduce thermal noise prevalent in ultrahigh-field fMRI scans and improve temporal signal-to-noise ratio (12, 13). The first two frames of each fMRI volume were removed for T1-equilibration effects. The anatomical and functional volumes were then preprocessed using Rodent Automated Bold Improvement of EPI Sequences (RABIES; (14)). Anatomical images were corrected for inhomogeneity and registered to the Dorr-Steadman-Ulman-Richards-Qiu-Egan (DSURQE) atlas (15–18). Functional volumes underwent slice-timing correction, realignment, registration to the anatomical image and subsequently to DSURQE atlas space. Functional images from the Columbia site were all resliced to 150 × 150 × 450 μm^3 to match data from the Technion site.

Following standard fMRI preprocessing, resting-state fMRI-specific preprocessing was performed using RABIES (confound correction step). A censoring mask was created based on a framewise displacement (FD) that exceeded 75 μm (including the frame prior and the two frames that followed each of the FD flagged frames). Simulated data of the censored frames were replaced with interpolated frames (Lomb-Scargle periodogram; (19)) and temporal filtering ($0.009 < f < 0.08$ Hz) was applied to the resultant time-series. In the final step, the censored frames were removed, and nuisance regression of the six motion parameters, ventricular, white matter, and global signals was performed. Images were spatially smoothed with a Gaussian kernel (full width half maximum [FWHM] of 0.5mm) following preprocessing.

Preprocessed timeseries were concatenated across sessions for each mouse at each age (mean % frames retained per mouse after motion scrubbing = 77%; std = 10%; range = 48%-93%). The amount of data across mice was equalized by sampling the same number of frames as were available for the mouse with the least amount of data, taken from the beginning of their timeseries (20). This process resulted in 2039 frames (~85 minutes) for each mouse imaged at the Technion site. As not all mice acquired at the Columbia site had 2039 frames, two separate versions of matrices (described below) were constructed for these animals: one version included only animals for which 2039 frames were present (n=18, 12 males); this version was used in comparisons including mice from both sites. To maximize the number of animals included in comparisons specific to the Columbia site (i.e., those conducted in Fig. 2B-D focusing on comparisons between 3 month old and 20 month old males and females), matrices and analyses were also constructed using all available frames for each animal (range=1456-2636 frames; mean=2117 frames; SD=352 frames). The comparisons and conclusions are comparable when limited to animals with 2039 frames.

MOUSE BRAIN NETWORK CONSTRUCTION

Network nodes

The Allen Institute Mouse Brain Common Coordinate Framework (CCFv3; (21)) was used to define network nodes. Adjustments to the CCFv3 parcel definitions were conducted as follows:

Neocortical adjustment: 86 Cortical regions from the CCFv3 were defined in a layer-nonspecific manner. To account for partial-volume effects from non-neuronal tissue, parcels were eroded from the cortical periphery by

creating an overlap mask of the average EPI and cortical layers 2-5 in the CCFv3 atlas; this mask was manually adjusted to ensure proper alignment. Parcels containing fewer than 14 voxels were excluded from the final node set.

Thalamic adjustment: 26 thalamic regions were defined in a similar manner using labels from the Allen Atlas. Due to the smaller nature of these parcels, no erosion was applied.

Hippocampal adjustment: The CCFv3 includes labels of the different hippocampal subfields. However, these labels contain both dorsal and ventral aspects of each subfield despite significant differences in functional connectivity patterns along the dorsal-ventral axis (4). To account for this known functional heterogeneity, we used a flattened surface-based representation of the dentate gyrus, CA3, CA1 and subiculum and divided each subfield into 5 segments along the dorsal-ventral axis, as previously described (4). These segments were then transformed from surface to volume, resulting in a total of 40 hippocampal nodes.

Striatal adjustment: Previous studies have demonstrated that the region labelled Caudoputamen in the CCFv3 can be further parcellated based on anatomical connectivity, and that the resultant parcels are characterized by distinct patterns of functional connectivity (8, 22). Therefore, we used normalized intensity from 16 anatomical tracing experiments conducted by the Allen Institute (**Table S1**) to divide the Caudoputamen labelled region into 6 subregions using a winner-takes-all approach. The 6 parcels represent frontal, somatomotor, lateral, auditory, visual, and medial striatal regions. The procedure was performed for the right hemisphere and parcels were then mirrored to the left hemisphere.

RSFC matrix construction

Each node's timeseries was computed by averaging across voxels within the node, yielding a n × t timeseries for each individual, where n is the number of nodes (156), and t is the number of frames. These timeseries were used to form a 156×156 matrix of Fisher's z-transformed Pearson's correlation values between each pair of nodes, and the diagonal of the matrix was set to 0.

Harmonization of matrices across acquisition sites

To account for differences in the protocol and head-coil used across acquisition sites, Correcting Covariance Batch Effects (CovBat, (23)) was employed to harmonize correlation matrices prior to statistical analysis. CovBat is designed to remove site effects in mean, variance, and covariance of correlation matrices while preserving effects of interest. Sex and age were held constant in the harmonization process, as these were variables of interest in the analysis. Accordingly, matrices were harmonized such that differences due to age and sex were preserved while differences due to site were minimized. As CovBat output is dependent on its inputs, the harmonization process was repeated for each version of matrices used in the analysis presented in the main paper and supplemental analyses (e.g., inclusion or exclusion of global signal regression, different numbers of frames contributing to matrix construction, etc.).

Assigning nodes to functional communities

Community detection: Community detection was performed using *Infomap* (24) on the mean of individual correlation matrices, across a range of edge densities (0.5-20%, increments of 0.5%). To account for differential correlation strengths, subcortical and cortical edges were thresholded independently, such that the suprathreshold edges from the cortex (78×156) and subcortex (78×156) were detected separately. This procedure is similar to existing analyses for integration of cortical and subcortical functional connectivity in humans (25, 26). Edges between nodes separated by less than 1.2mm (just over 2x the smoothing kernel) from center-to-center (defined as the Euclidean mean of all voxel coordinates within a node) were excluded from community detection (i.e., these edges were set to 0) to avoid spatial autocorrelation confounds, again mirroring work in humans (27). The algorithm was repeated 100x, and the mode assignment for each node was used as a final label at each density. As an alternative approach for identifying a consensus set of labels across

repetitions, the normalized mutual information (NMI) was computed between each of the 100 assignments at each density, and the assignment with highest mean NMI was selected as the consensus labelling for that density. These two approaches yielded the same final assignments.

"Sparsest" density community labelling algorithm: At each density, Infomap outputs a 1 × 156 vector of community labels (one label for each node in the correlation matrix). To condense labels across densities, a previously used algorithm (see: (28)) was applied to this output. Briefly, given a starting density (beginning at the sparsest density, e.g., 0.5%), nodes were assigned to a community if the Infomap community assignment at that density included at least 2 nodes. If a node was the only member of its assigned community, it remained unlabeled, and the next-sparsest density was evaluated. Previously unassigned nodes were iteratively assessed at increasing densities and assigned a final community label if their Infomap label was shared by at least 1 other node. This process was repeated until either all nodes belonged to a community or the highest examined density (i.e., 20%) was reached. Any nodes remaining unassigned at the highest density were labeled as such and excluded from further analysis. Thus, the final community label was based on assignments evaluated across a range of densities, where a node's community label was based on the sparsest density at which it belonged to a community with multiple nodes. The final community labels yielded by this approach were thus sensitive to the starting density (i.e., the sparsest density evaluated) as well as the highest density examined (which was held constant at 20% in the present analysis). This process is highlighted in **Fig. S3**. When we refer to a community assignment being at a certain density in the present work, we are referring to the starting density assessed in the algorithm.

HUMAN DATA ACQUISITION, PROCESSING, AND NETWORK CONSTRUCTION

Human datasets

HCP Development (HCP-D). The dataset includes data collected from 652 participants (age range: 5–22 years; HCP-D release 2.0 dataset [https://nda.nih.gov]). The scanning protocol was approved by the Washington University in St. Louis's Human Research Protection Office and all participants provided written informed consent. Participants over 18 years old with at least 20 min of clean resting-state data (see RSFC Preprocessing) were included in the final sample (N = 122, age range: 18-22 years, M = 20.13 years, SD = 1.20).

HCP Young Adults (HCP-YA). The dataset includes data collected from 1206 participants (age range: 21–37 years; HCP s1200 release dataset (29, 30)). This release has been made publicly available (http://www.humanconnectome.org). The scanning protocol was approved by the Washington University in St. Louis's Human Research Protection Office and all participants provided written informed consent. Participants with at least 20 min of clean resting-state data (see RSFC Preprocessing) were included in the final sample (N = 729, age range: 22-37 years, M = 28.76 years, SD = 3.74).

HCP Aging (HCP-A). The dataset includes data collected from 725 participants (age range: 35–100 years; HCP-A release 2.0 dataset [https://nda.nih.gov]). The scanning protocol was approved by the Washington University in St. Louis's Human Research Protection Office and all participants provided written informed consent. Participants under 90 years old with at least 20 min of clean resting-state data (see RSFC Preprocessing) were included in the final sample (N = 328, age range: 36-90 years, M = 57.16 years, SD = 14.77).

Human fMRI acquisition

Participants in HCP-YA underwent imaging on a customized Siemens 3T Skyra scanner using a 32-channel head coil at Washington University in St. Louis. Participants in HCP-A and HCP-D underwent imaging on a Siemens 3T Prisma whole-body scanner (Siemens, Erlangen, Germany) with a Siemens 32-channel head coil at one of 4 different sites (Massachusetts General Hospital, University of California-Los Angeles, University of Minnesota, and Washington University in St. Louis). Each participant completed two scanning sessions on two separate days. Only de-identified data were downloaded and used in the current study.

Anatomical Images. T1-weighted magnetization-prepared rapid acquisition gradient echo (MP-RAGE) structural scans (TR = 2500 ms, TE = [HCP-YA: 2.1 ms; HCP-A/D = 2.22 ms], TI = 1000 ms, resolution = [HCP-YA: 0.7mm

isotropic voxels; HCP-A/D = 0.8mm isotropic voxels], flip angle = 8°) and T2-weighted structural scans (TR = 3200 ms, TE = [HCP-YA: 565 ms; HCP-A/D: 563ms], resolution = [HCP-YA: 0.7mm isotropic voxels; HCP-A/D = 0.8mm isotropic voxels]) were acquired.

Functional Images. Resting-state functional MRI images were acquired while the participants fixated on a white crosshair on a black background using a gradient-echo EPI sequence (multiband factor = 8, TR = [HCP-YA: 720 ms; HCP-A/D: 800 ms], TE = [HCP-YA: 33.1 ms; HCP-A/D: 37 ms], flip angle = 52°, 104 × 90 matrix size, 72 slices, 2 mm isotropic voxels, and 1200 time points [14.4 min] per scan of HCP-YA, and 488 time points [6.5 min] per scan in HCP-A and HCP-D). There were 2 resting-state scans in each scan session, with different phase-encoding directions (HCP-YA: RL and LR; HCP-A: AP and PA; HCP-D: AP and PA) in each scan.

Processing of anatomical MRI image and cortical surface

Anatomical MRI images were processed using HCP Pipeline structural processing that consists of 3 parts (PreFreeSurfer, FreeSurfer and PostFreeSurfer). The PreFreeSurfer pipeline produced an undistorted native structural volume space for each participant, aligned the T1-weighted and T2-weighted images, performed a B1 (bias field) correction, and registered the participants' native structural volume space to MNI space. The FreeSurfer pipeline (version 6.0) segmented the volume into predefined structures, reconstructed pial and white matter cortical surfaces, and performed FreeSurfer's standard folding-based surface registration to the fsaverage surface atlas. The PostFreeSurfer pipeline output final NIFTI volume and GIFTI surface files, applied surface registration to the Conte69 surface template (31), down-sampled registered surfaces, and created the final brain mask.

Basic fMRI processing

All HCP datasets employed the HCP Pipeline's fMRI Volume processing to correct gradient-nonlinearity-induced distortion, realign the timeseries (EPI volumes) to correct for head motion, perform EPI fMRI image distortion correction due to phase encoding directions, and combine all of the transforms for each registration and distortion correction step into a single nonlinear transformation that can be applied in a single resampling step. After transforming the fMRI volumes, the intensity across runs was normalized to a whole brain mode value of 1000 (32).

Resting-state functional correlation (RSFC) preprocessing

Additional preprocessing steps were taken to reduce spurious variance unlikely to reflect neuronal activity in RSFC data (33). (i) Data were demeaned and detrended. (ii) Multiple regression of the BOLD data was performed to remove variance related to the whole brain gray matter signal (defined by each participant's own anatomy), ventricular signal, white matter signal, six detrended head realignment parameters obtained by rigid-body head motion correction, and the first-order derivative terms for all aforementioned nuisance variables. While the use of global signal regression in RSFC processing has been a subject of differing perspectives, it is objectively effective for minimizing motion-related artifacts in humans (34, 35), and the recommended method to reliably remove global respiration-related artifacts when direct estimates of respiration are unavailable (36). Because older adults are more prone to head movement (37, 38) that leads to altered RSFC profiles (19, 35), it is critical to minimize the source of bias that may contribute to erroneous estimation of RSFC. (iii) To reduce the effect of motion artifact on RSFC, data were processed following a "scrubbing" procedure (19). Motion-contaminated volumes were identified by frame-by-frame displacement (FD) that was calculated as the sum of absolute values of the differentials of the 3 translational motion parameters and 3 rotational motion parameters (19). Recent studies demonstrated that high-frequency respiratory artifact can confound estimates of FD, in particular for scans using multiband sequences with short TRs (e.g., (39)). As such, the motion parameters were filtered to remove high-frequency components (>0.1Hz) prior to the estimate of FD and a more stringent cutoff of 0.04mm was used for FD. In addition, data between two motion-contaminated frames that were fewer than 5 frames apart were also flagged. These flagged motion-contaminated frames were removed and interpolated for the

subsequent processing. (iv) Band-pass filtering (0.009Hz < f < 0.08Hz) was performed. (v) Interpolated frames that were used to preserve the time series during regression and bandpass filtering were removed.

Surface mapping and CIFTI generation

Connectivity Informatics Technology Initiative (CIFTI) format files were generated using HCP Pipeline fMRI Surface processing to integrate information from all possible brain ordinates, including the cerebral cortex, subcortical structures, and the cerebellum. To do so, the timeseries data of the cortical surface were derived by resampling functional volumes to 32k mesh surface using single deformation maps derived from surface data processing and smoothed with a 2mm FWHM Gaussian kernel. The subcortical data were smoothed in volumetric space with a 2mm FWHM Gaussian kernel. Finally, the timeseries data of the cortical surface and volumetric time series of FreeSurfer-labeled subcortical structures and the cerebellum were combined to create CIFTI files (29).

Frame equating

The first 20 minutes of motion-scrubbed resting-state data was used to construct RSFC matrices for each human individual. Since different HCP datasets have different TRs, the number of frames equal to 20 minutes varied (1667 frames for HCP-YA, 1500 frames for HCP-A/HCP-D).

RSFC matrix construction and community assignment

The Schaefer 400 parcellation (40) and “Kong2022 17 network” community label ((41); created using HCP participants) set were used for human network analysis. While age-related system segregation decline is found across a variety of nodes and community labels (42–44), a key consideration here was to use a label set defined based on HCP data, since the mouse community labels were also derived in-sample. As such, we used the Kong labels given that they were defined from HCP-YA data, thus increasing comparability of procedures used across the two species. Node-level timeseries extraction and matrix construction were conducted similarly to the mouse procedures described above.

Harmonization across acquisition sites

As HCP-YA used a different MRI scanner and scanning parameters from HCP-D and HCP-A, harmonization is necessary to combine the three datasets. CovBat (23) was employed for this purpose. Rather than targeting site effects for harmonization as with mice, study effects were targeted, as acquisition differences between HCP-YA and the other HCP datasets represented the greatest sources of variance to be harmonized. Age and sex were held constant to preserve differences due to these factors.

BRAIN NETWORK ANALYSIS

System segregation calculation

System segregation was calculated on the weighted matrices. As the interpretation of negative resting-state correlations is somewhat ambiguous due to necessary preprocessing procedures (45, 46), negative values for each human RSFC matrix were set to zero prior to system segregation calculation. For mice, systems with fewer than 4 nodes were excluded from system segregation calculation to avoid biased estimates of within- and between-system correlation in small systems, and negative edges were set to 0. Notably, this step did not result in exclusion of any nodes at the density used in the primary analysis in mice (7%). Since the minimum system size in the human systems atlas was already greater than 4 nodes, exclusion of smaller systems was not necessary for human data. System segregation quantifies the extent to which brain networks are clustered into distinct systems, according to the following equation:

$$system\ segregation = \frac{\frac{\sum_{w}^{W} Z_w}{W} - \frac{\sum_{b}^{B} Z_b}{B}}{\frac{\sum_{w}^{W} Z_w}{W}}$$

where Z_w represents Fisher's z-transformed correlation values between nodes in the same system, Z_b represents correlation values between nodes belonging to different systems, W represents the total number of within-system edges in the network, and B is the total number of between-systems edges in the network. Thus, system segregation measures the relative magnitude of within-system edges compared with between-system edges (42).

Analysis of system segregation in mice

In all primary analyses, mouse system segregation values were calculated based on communities derived from the group average matrix of young adult (3-4 months old) mice (as described above) from 1-10% edge density, in increments of 1%. Results reported in the main text are based on communities detected at 7% edge density and results using alternate densities are presented in **Fig. S9.**

To estimate slopes of system segregation differences and changes across adulthood, a linear mixed effects model was applied using system segregation values from all animals. The model was implemented using the *fitlme* function in MATLAB 2019 with the following formula:

system segregation ~ age + sex + (1 + age l individual),

Where individual encodes individual mouse IDs, to account for the subset of mice that were imaged longitudinally.

An age-by-sex interaction term was initially included in the linear model. However, there was no significant interaction effect, and the total variance explained by the model was comparable to the above model without the interaction term. The lack of an age-by-sex interaction was consistent with results reported in **Fig. 2D**. As such, the interaction term was removed to minimize likelihood of false negatives and positives of the remaining main effects in the model (47).

Analysis of system segregation in humans

Human system segregation was calculated based on systems labelled based on a set of pre-defined communities (Kong17 from (41)) for each individual matrix in the combined HCP-D/HCP-YA/HCP-A dataset.

Replicating previous work (20, 42), a relationship between age and system segregation was tested for by calculating Pearson's correlation.

Cross-species comparison of system segregation

Differences in system segregation were compared across mice and humans. All mouse cohorts were used to maximize the set of ages compared with human data. For mice, communities based on the young adult group average were used for system segregation calculation in order to ensure comparability to human network comparisons which were also based on a younger adult atlas. Human ages were aligned with mouse ages according to approximate benchmarks which are based on tract-tracing and gene expression, (48) as well as a set of additional biomarkers including but not limited to T-cell subsets, tail length, hair regrowth, wound healing, coordination and strength, collagen denaturation, and lifespan (49). To account for uncertainty in exact cross-species age correspondence, a range of alignments were examined (**Fig. S15**). A linear mixed effects model was used to test comparisons across human and mouse data with the following formula:

system segregation ~ age + species + sex + age*species + (1 | individual),

where individual encodes individual IDs to account for mice imaged longitudinally.

Cross-species comparison of within- and between-system RSFC

Species-related comparisons of within- and between-system RSFC focused on mean young adult matrices (parallel comparisons were also conducted on older adult matrices in **Fig. S16**). For mice, this group average matrix was based on all young adult mice (3-4 months). For humans, this group average matrix was based on all HCP-YA data (22-35 years). As edge weights were systematically greater in humans than mice, matrices were normalized for each species prior to statistical testing by dividing each edge by the mean edge weight for that species.

For comparisons of between-system connection distances, for each species, edge distance was categorized as short-range or long-range, relative to half the maximum Euclidean distance between centers of network nodes.

2. SUPPLEMENTARY RESULTS

2.1 – *Specificity of mouse RSFC patterns using left hemispheric seeds.*

The specificity analysis reported in main text **Fig. 1** used seed regions (VISp and SSp-bfd) in the right hemisphere. We also tested whether the observations hold using seed regions in the left hemisphere**.** RSFC maps for these seed regions were compared both at the group average level and within individual mice; group average seed-based correlation maps reveal distinct RSFC topographies for each seed location (**Fig. S1A**). As the sets of regions that share functional similarities with these seed regions are well-defined (visual regions, denoted as VISxx and comprising CCFv3 parcels with the label prefix "VIS", and primary somatosensory regions, denoted as SSp-xx and comprising CCFv3 parcels with the label prefix "SSp"), we evaluated specificity of RSFC by comparing correlations between each seed region and these two sets of target regions. We tested whether visual regions are preferentially correlated with VISp relative to SSp-bfd and whether primary somatosensory regions are preferentially correlated with SSp-bfd relative to VISp**.** Further, we evaluated specificity of RSFC for these systems at two levels: by examining RSFC to parcels within each system (group-level analysis) as well as RSFC to the system as a whole (aggregating across constituent parcels; individual-level analysis).

First, group average RSFC seedmap values were extracted for every parcel labeled as "VIS" or "SSp" in the CCFv3, yielding four sets of connectivity groupings (VISp to VISxx, VISp to SSp-xx, SSp-bfd to VISxx, and SSp-bfd to SSp-xx). Visual regions exhibit greater functional connectivity with VISp, and somatosensory regions exhibit greater functional connectivity with SSp-bfd (**Fig. S1B**). A linear mixed effects model testing for an interaction between seed-target pairs confirmed this regional dissociation ($F(1,56)=20.095$, $p<.001$). Planned comparison paired t-tests revealed significantly stronger functional connectivity between VIS targets and VISp compared with SSp-bfd ($t(12)=3.519$, $p=.004$), and significantly stronger functional connectivity between SSp targets and SSp-bfd compared with VISp ($t(16)=3.159$, $p=.006$).

The dissociation between seed regions and their respective visual and somatosensory targets is also evident in seedmaps of individual mice rather than group average seedmaps (**Fig. S1C**). Whereas the group average analysis examined RSFC between seeds and each of the target regions individually, this second analysis examined RSFC between seed regions and the entire set of target regions (VISxx or SSp-xx). To test for this circuit-level specificity, the mean functional connectivity for each seed-target grouping was calculated for each individual mouse and compared in a 2×2 repeated-measures ANOVA that tested for an interaction in correlation strength between seed-target pairs (VISp to VISxx, VISp to SSp-xx, SSp-bfd to VISxx, and SSp-bfd to SSp-xx; VISxx/SSp-xx denote the inclusion of all parcels with the VIS/SSp prefix, excluding VISp/SSp-bfd, respectively; **Fig. S1C**). This analysis revealed a significant interaction between seeds and targets ($F(1,30)=500.47$, $p<.001$). Planned comparison paired-samples t-tests confirmed that across subjects, the VIS target region set is more strongly correlated with VISp compared with SSp-bfd ($t(30)=19.785$, $p<.001$), and the SSp target region set is more strongly correlated with SSp-bfd compared with VISp ($t(30)=15.161$, $p<.001$).

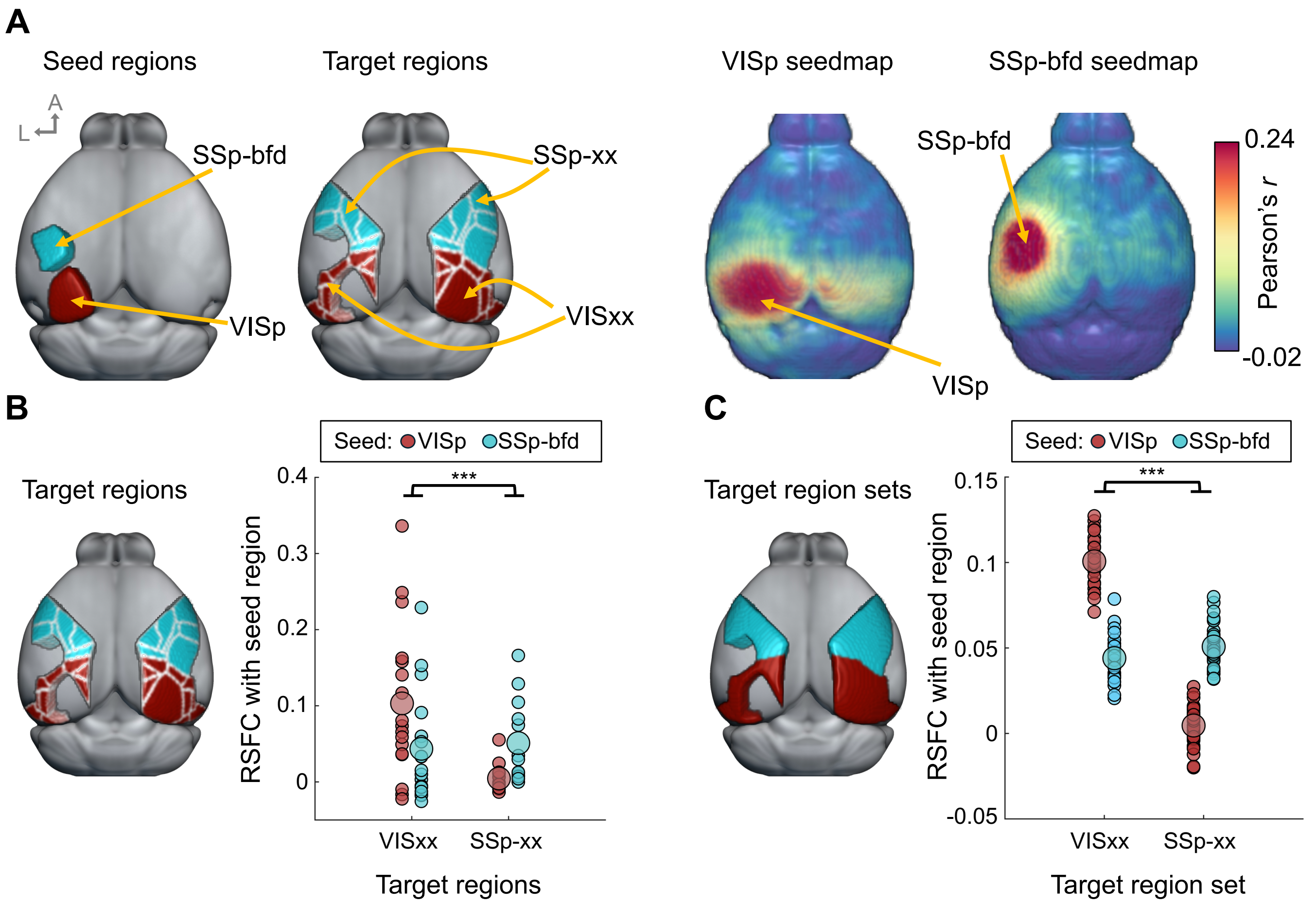

Supplementary Fig 1. Specificity of mouse RSFC patterns is evident using left hemisphere seed regions. The specificity analysis from main text **Fig. 1** is replicated using seeds in the left hemisphere. **(A)** Resting-state functional correlation (RSFC) seedmaps were compared for two neighboring cortical regions to test the validity and specificity of functional connections. From left to right: left hemisphere primary visual cortex (VISp) and barrel cortex (SSp-bfd) parcels were used as RSFC seeds. Target regions were defined as CCFv3 parcels labeled as vision-related (VISxx; red) or primary somato-sensory (SSp-xx; blue). Seed-correlation maps depict strengths of correlations across the cortex for each of the two seed regions. Despite proximity of VISp and SSp-bfd seeds, RSFC seedmaps for the two regions are topographically distinct and differ in their extent of contralateral (homotopic) functional connectivity. **(B)** Functional connectivity between seed and target regions was quantified by extracting group average seedmap correlation values for each target region, in order to test whether VISxx targets are preferentially correlated with the VISp seed region, and whether SSp-xx targets are preferentially correlated with the SSp-bfd seed region. Each dot in (B) corresponds to a target region, where the dot's value reflects the mean correlation between that target region and a given seed region. The brain image depicts the target regions used to extract mean correlations (i.e., parcels). **(C)** Specificity of VISp and SSp-bfd functional connectivity is present across individual mice. The brain image depicts the two target region sets used to extract mean correlations. When evaluated as a set of functionally related regions across mice, the VISp seed exhibits stronger RSFC with the VISxx target region set compared to the SSp-bfd seed. Conversely, the SSp-bfd seed exhibits stronger RSFC with the SSp-xx target region set compared to the VISp seed. Each dot corresponds to an individual mouse, where the value reflects the mean correlation between each target region set and a given seed region. In (B) and (C), larger dots represent group means, as a visual aid. ****p<.001*

2.2 – Regions exhibit strong homotopic resting-state functional correlations.

Measurement of homotopic RSFCs has been previously used to evaluate the validity of resting-state correlations in mice (6, 50). Homotopic cortical areas are often linked by direct structural connections (51), and strong homotopic RSFCs have been reliably detected in awake humans (52), and sedated non-human primates (53) and mice (54). We evaluated whether homotopic regions exhibit greater RSFC compared to functional connectivity among other sets of contralateral and ipsilateral brain regions, using the resting-state data collected in awake young adult mice (3-4 months old). For each cortical parcel, three measures were extracted: homotopic FC, contralateral FC, and ipsilateral FC. Homotopic FC was defined as a given seed parcel's RSFC to the contralateral parcel with the same CCFv3 label (e.g., left hemispheric VISp is homotopic to right hemispheric VISp). Contralateral FC was defined as the median RSFC between a given seed parcel and all non-homotopic contralateral cortical parcels. Ipsilateral FC was defined as the median RSFC between a given seed parcel and all ipsilateral cortical parcels. These three measures were computed separately for every parcel based on each animal's RSFC network matrix and then averaged across animals, yielding one parcel-level estimate of each measure (i.e., for each parcel, the median ipsi/contralateral FC is computed for each animal, and these median values are then averaged across animals). Homotopic FC exceeds contralateral FC for 77/78 parcels; a paired samples t-test confirmed higher homotopic FC than contralateral FC ($t(77)=9.2$, $p<.001$). Homotopic FC exceeded ipsilateral FC for 65/78 parcels; a paired samples t-test confirmed higher homotopic FC than ipsilateral FC ($t(77)=3.48$, $p<.001$).

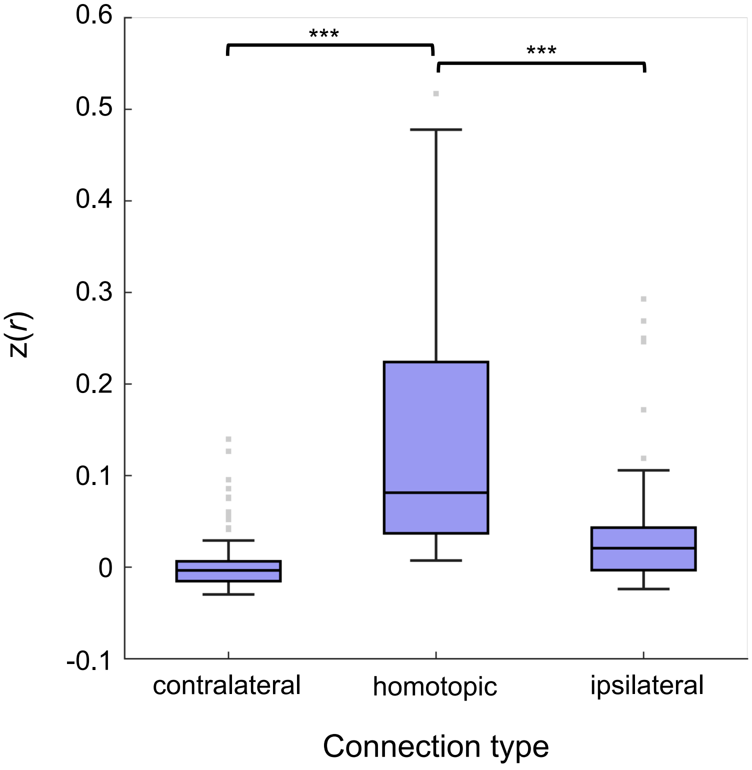

Supplementary Fig 2. In mice, homotopic regions exhibit stronger resting-state functional correlations compared to functional relationships with other contralateral or ipsilateral regions. Boxplots show distribution of (from left to right) median FC between seed parcels and all non-homotopic contralateral parcels, FC between seed parcels and homotopic parcels, and median FC between seed parcels and all non-seed ipsilateral cortical parcels. All values represent the parcel-level measure (median contralateral FC, homotopic FC, or median ipsilateral FC), averaged across animals. Homotopic RSFC exceeds both contralateral RSFC and ipsilateral RSFC. ***$p<.001$.

2.3 – *Nodes and communities included in system segregation calculation across edge densities*

The consensus community assignment algorithm applied to Infomap output assigns nodes to communities at varying matrix edge densities (see main text Methods). Nodes that are either not assigned to a community (e.g., isolated nodes) or nodes that belong to a community with 3 or fewer nodes are excluded from analyses (and consequently system segregation calculation); the proportion of nodes excluded varies across edge densities examined. For communities based on 7% edge density (i.e., the community labels used in the main analysis), all nodes were assigned to a community (**Fig. S3A**). In addition, while many communities are excluded for being too small (composed of 3 or fewer nodes) at the lowest densities, all communities included at least 4 nodes at 7% density (**Fig. S3B**).

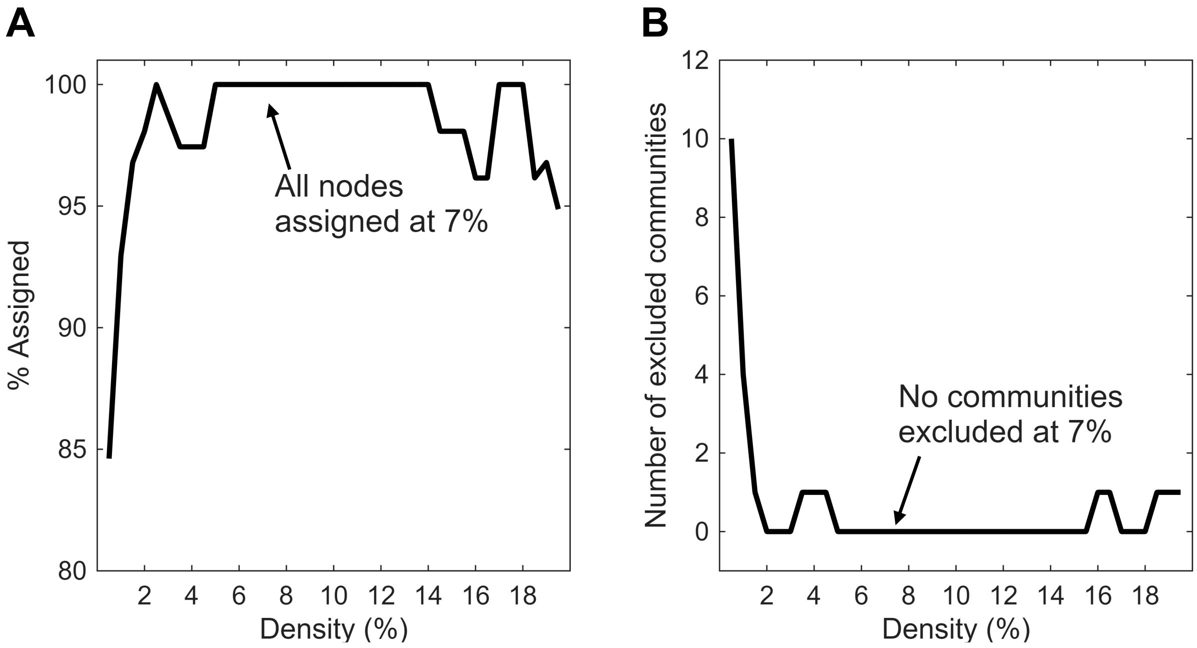

Supplementary Fig. 3. Labelling of nodes and communities across edge density values. (A) The number of nodes assigned to a final community label varies across edge densities, but all nodes were assigned at the density used in the primary analysis (7%). **(B)** Communities with 3 or fewer constituent nodes are excluded from system segregation calculation. At 7% edge density, no communities were excluded.

2.4 – Comparison of resting-state systems (community organization) with structurally-defined system labels.

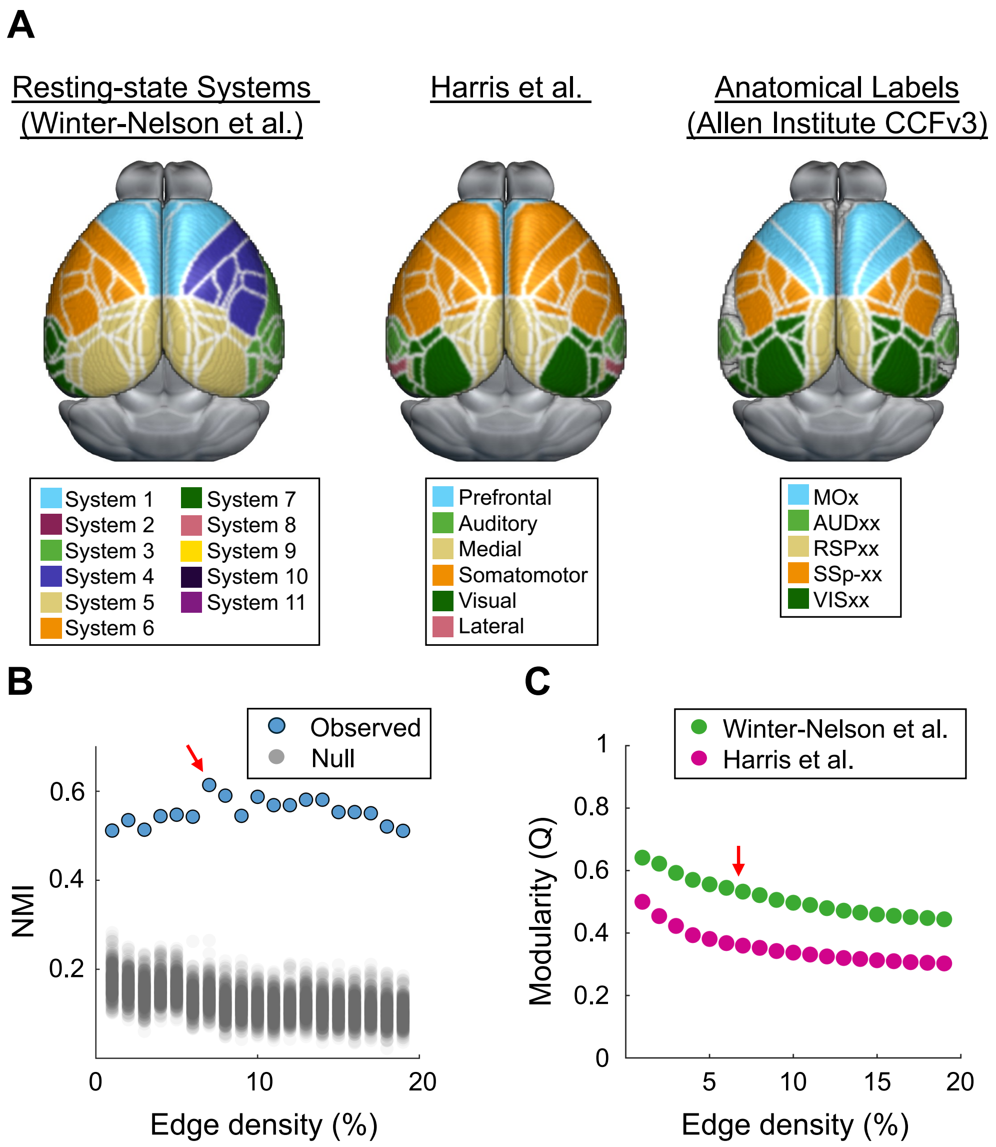

Supplementary Fig. 4. Comparison of resting-state systems (community organization) with structurally-defined system labels. The system labels (community assignments) generated in the current work align in part with established structure-based system labels, but exhibit notable differences. **(A)** Left: Systems used in the main text from 3-4 month old mice (Resting-state systems [Winter-Nelson et al.], based on 7% edge density), middle: a previously published set of cortical system labels based on synaptic tracer experiments in 2-3 month old mice ('Harris'; (55)), right: anatomical labels of selected parcels provided with the CCFv3 atlas, grouped and labelled by their atlas names ('Anatomical labels'; (21)). Anatomical labels are grouped as follows: Motor (MOx; light blue), Primary somatosensory (SSp-xx; orange), Retrosplenial (RSPxx; beige), Visual (VISxx; dark green), and Auditory (AUDxx, light green). CCFv3 parcel outlines are overlaid on each of the brains as an aid to compare system topographies. Visually, it can be appreciated that the resting-state systems align with the structurally defined systems (Harris labels), although some structurally-defined systems are merged in the functional systems (e.g., the retrosplenial [RSPxx] and visual [VISxx] parcels form a single system) and other structural systems are divided across functional systems (e.g., the Harris communities merge motor [MOx] and primary somatosensory [SSp-xx] parcels, while the functional systems separate them into distinct systems). **(B)** The similarity of system labels defined from resting-state and structural data is confirmed statistically by computing normalized mutual information (NMI) between the functionally defined system labels (based on 7% edge density) and the Harris system labels. Given that RSFC-based system labels can be generated across a range of edge densities, NMI was computed between Harris labels and RSFC-based labels derived from the present dataset

by calculating communities based on thresholds ranging from 1-20% edge density. The red arrow denotes the comparison using systems defined at 7% density. As Harris labels are restricted to the cortex, this comparison is limited to cortical parcels. A null distribution was created by computing NMI between the RSFC-based systems and 100 random reshufflings of the Harris system labels. NMI between the two label sets exceeds the null distribution across all edge densities tested based on one-sample t-tests ($p<.001$ for all densities). **(C)** Modularity (Q; (56)) is a measure used to estimate the extent to which a given set of system labels partitions a network into distinct modules (i.e., communities). RSFC system labels exhibit higher modularity values of the mouse functional connectome compared with the Harris system labels. As modularity of resting-state systems is calculated on a thresholded matrix (matching the threshold at which communities are defined, ranging from 1-20%), modularity estimates were generated for Harris system labels applied to the mean young adult resting-state matrix across the same range of densities in order to yield comparable results across label sets. As with the NMI analysis, modularity was computed based only on parcels that are common to both label sets. The red arrow denotes Q-values based on 7% density.

2.5 – Comparison of community organization with alternate functionally-defined community labels.

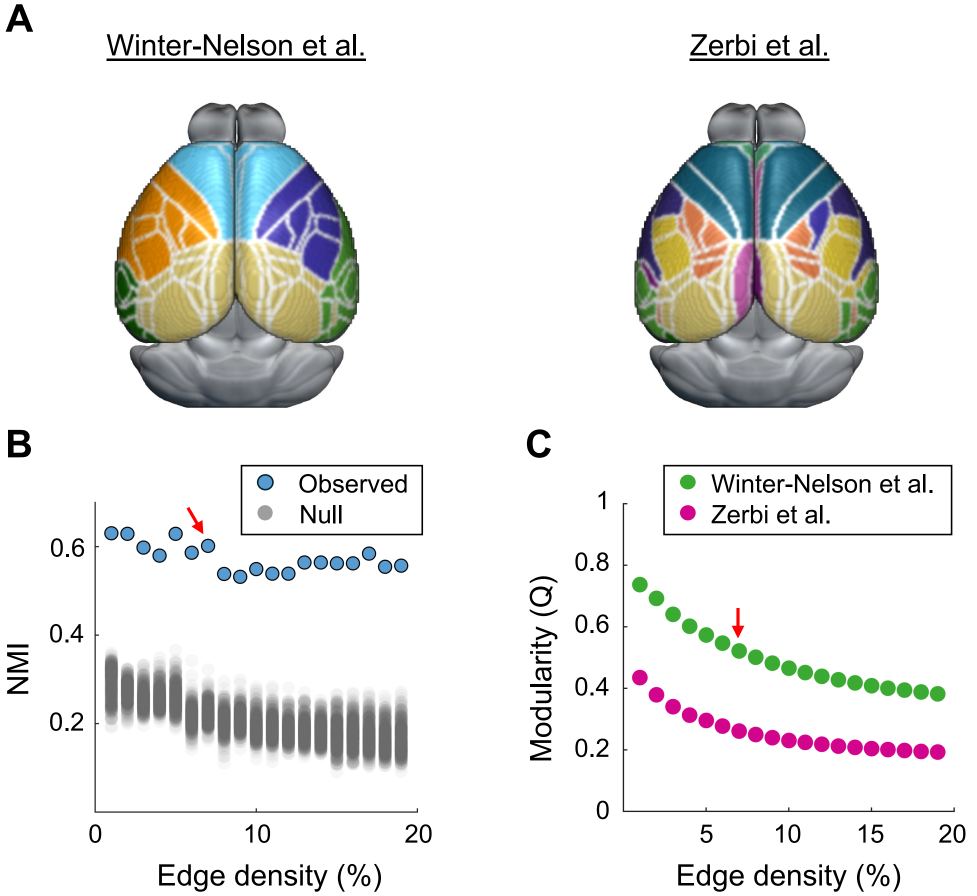

Supplementary Fig. 5. Comparison of community organization with alternate functionally-defined community labels. The community labels generated in the present work align in part with previously established RSFC-based communities, but again exhibit meaningful differences. **(A)** Left: Systems used in the main text from 3-4 month old mice (Winter-Nelson et al., based on 7% edge density), right: a previously published set of cortical community assignments derived from resting-state fMRI data in 3 month old mice, which was collected under anesthesia ('Zerbi'; (57)). Since the Zerbi communities are based on independent components analysis at the voxel level and thus do not map directly to specific CCFv3 parcels, a winner-take-all approach was used to assign parcels to a Zerbi community based on mean component weight within each parcel. CCFv3 parcel outlines are overlaid on each of the brains as an aid to compare system topographies. From this visualization, it can be appreciated that the two label sets overlap in several locations (e.g. similar motor systems) but also exhibit differences (e.g., lateralized vs. bilateral somatosensory systems), **(B)** The similarity of labels is confirmed statistically by computing normalized mutual information (NMI) between the Winter-Nelson system labels (based on 7% edge density) and the Zerbi system labels. As RSFC-based system labels can be generated across a range of edge densities, NMI was computed between Zerbi labels and RSFC labels derived from the present dataset by calculating communities on matrices ranging from 1-20% edge density. The red arrow denotes the comparison using systems defined at 7% density. As Zerbi et al. include subcortical areas, this comparison included the full set of 156 parcels. To form a null distribution, Zerbi system assignments were randomly shuffled 100 times prior to NMI calculation. NMI between the two label sets exceeds the null distribution across all edge densities tested based on one-sample t-tests (p<.001 for all densities). **(C)** The system labels derived from the present dataset exhibit higher modularity values of the mouse functional connectome compared with the Zerbi system labels, although it should be noted that the Zerbi system labels were defined out-of-sample. As modularity of resting-state systems is calculated on a thresholded matrix (matching the threshold at which systems are defined, ranging from 1-20%), separate modularity estimates were generated for Zerbi labels applied to the mean young adult resting-state matrix across the same range of densities in order to yield comparable results across

label sets. As with the NMI analysis, modularity was computed based on all parcels. The red arrow denotes Q-values based on 7% density.

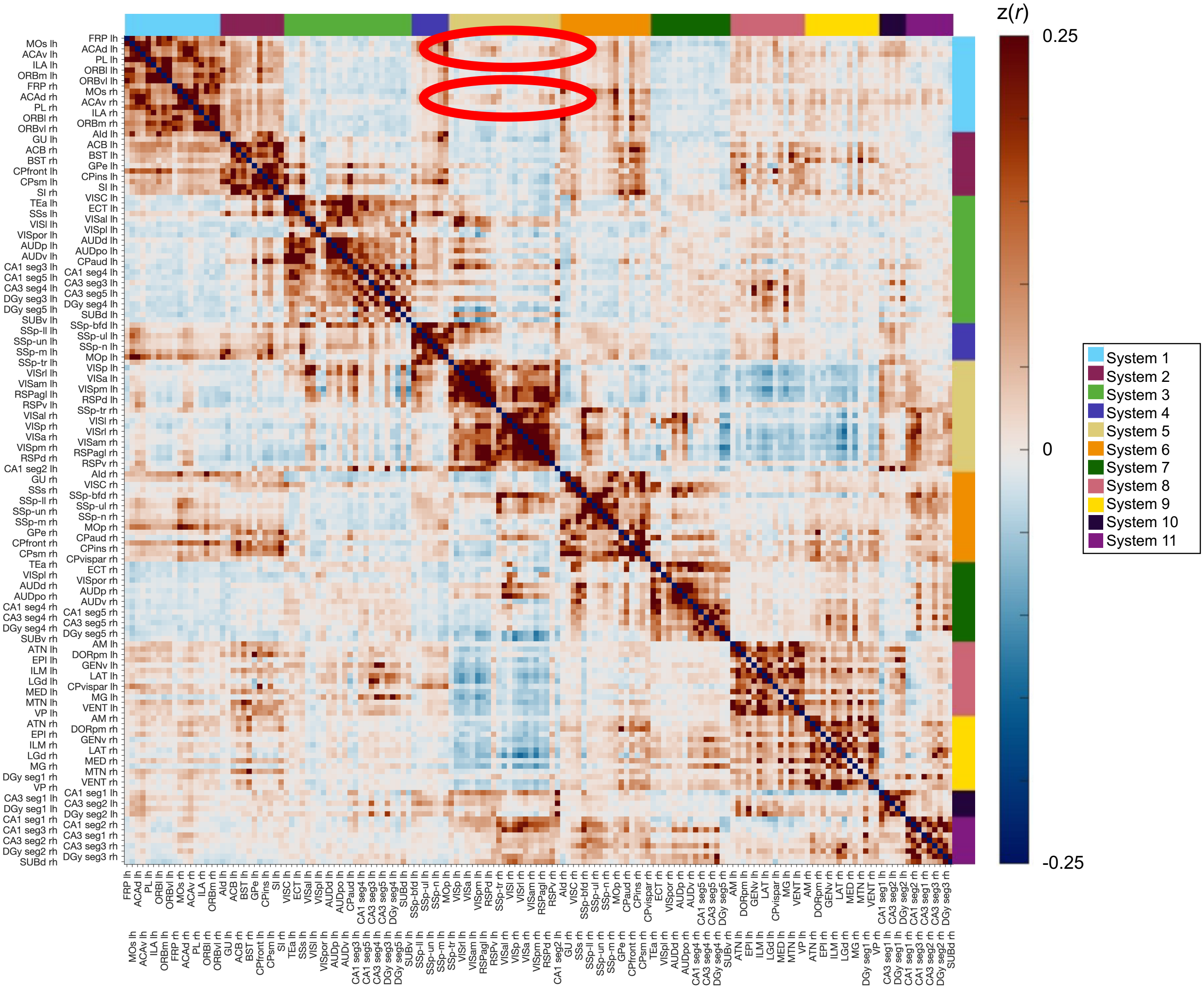

2.6 – Modular functional organization of the mouse brain.

Supplementary Fig. 6. Modular functional organization of the mouse brain.
The young adult (3-4 month old) mean RSFC matrix used for community detection is shown here with regions (nodes) labeled according to their names from the CCFv3 annotated atlas (21); node labels are staggered to improve legibility. Nodes are sorted according to community assignments (functional systems) reported in the main text (**Fig. 2A**). In addition to the modular architecture delineated by system labels, additional interareal relationships not captured by the modular structure are also apparent in this visualization. A particular circuit of interest, sometimes described as a potential analogue of the human default mode network (58), is denoted by red circles. This circuit includes anterior cingulate regions (ACAv, ACAd) and posterior regions including retrosplenial (RSPxx) and visual (VISxx) regions (58, 59). While there exist several region-to-region correlations with negative RSFCs between System 1 (light blue) and System 5 (beige), positive RSFCs exist between the anterior cingulate regions of System 1 and retrosplenial and visual regions of the System 5, consistent with the previously described medial circuit (58).

2.7 – Age-related declines in RSFC system segregation using age-group specific system labels.

A possible explanation for age-related declines in system segregation is that the brain's community organization differs in older adulthood, and that differences in segregation are due to their misalignment with the young-adult defined community atlas rather than a global dedifferentiation of the functional systems. Previous work from members in our group has demonstrated that in humans, while cortical parcellations and communities are slightly modified over the course of adulthood, declining system segregation is still evident when accounting for these modifications (44). Here, we adopt a similar approach to compare system segregation of younger (3-4 months old, 6 males, 6 females) and older adult (20 months old, 9 males, 9 females) mice from the Columbia site: community detection was conducted separately for each age group, based on the mean RSFC matrix of in-sample younger and older adult mice, and system segregation values were calculated based on age-specific communities applied to the corresponding mice. Communities were defined based on 7% edge density.

To evaluate differences in within-system and between-system correlations between the four groups (young adult females, young adult males, older adult females, and older adult males), 2×2 ANOVAs were run to test for main effects of age, sex, and their interaction on mean within- and between-system RSFC (**Fig. S7A**). Within-system connectivity did not show any significant differences between groups (age: $F(1,26)=0.006$, $p=.941$; sex: $F(1,26)=0.329$, $p=.571$; age-by-sex interaction: $F(1,26)=1.503$, $p=.231$), while between-system connectivity increased significantly between younger and older mice ($F(1,26)=7.988$, $p=.009$). Between-system connectivity did not differ between sexes ($F(1,26)=0.202$, $p=.657$), nor was there an age-by-sex interaction ($F(1,16)=0.007$, $p=.932$). A 2×2 ANOVA comparing system segregation of the four groups (**Fig. S7B**) revealed a significant effect of age wherein older mice had lower system segregation than younger mice ($F(1,26)=16.995$, $p<.001$), but no effect of sex ($F(1,26)=1.017$, $p=.323$) or age-by-sex interactions ($F(1,26)=0.557$, $p=.462$). To summarize, the results presented in the primary analysis (**Fig. 2C-D**) are consistent when using age-group specific system labels.

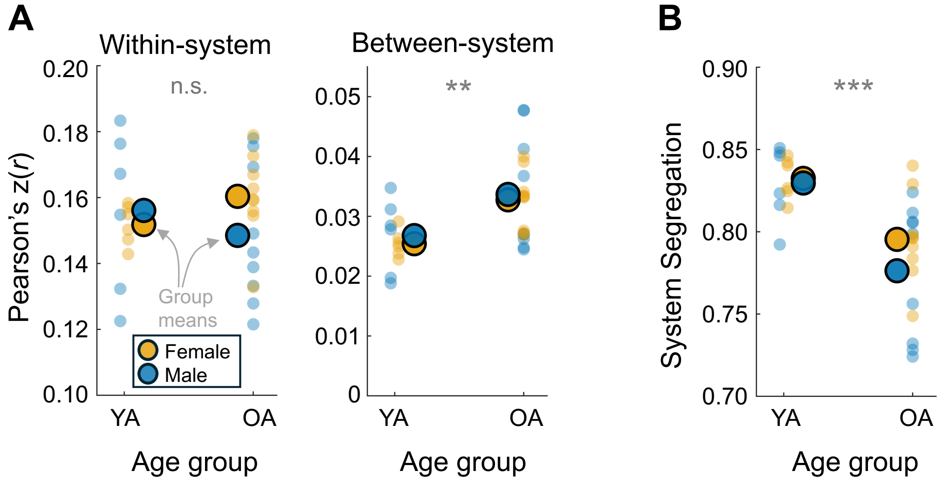

Supplementary Fig. 7. Age-related declines in RSFC system segregation using age-group specific system labels. (A) Within-system RSFC does not differ across age groups, although between-system RSFC is greater in older-age adult mice. The differences did not vary by sex. **(B)** Younger mice have higher system segregation than older mice when using age group-specific system labels. These effects are consistent between male and female mice. YA: young adults, OA: older adults. ***p*<.01, ***p<.001

2.8 – Age-related RSFC network alterations are evident within and between several systems.

To explore which specific interareal relationships differ between younger and older adult mice, 2-sample t-tests of all RSFC matrix edges were performed to compare groups of younger versus older mice scanned at the Columbia site (i.e., the data used in main text **Fig. 2B-D**), revealing the strength and direction of age-related differences for each pairwise functional relationship. Resulting p-values were Benjamini-Hochberg FDR corrected using the MATLAB 2019 *mafdr* function. First, it can be appreciated from **Fig. S8** that edge-level differences are grouped by system assignments: edges between nodes in the same systems tend to exhibit similar directions of age-related differences. In addition, the increases of between-system RSFC in older age animals is evident among several systems (blue colors in the off-diagonal blocks in the matrices depicted in **Fig. S8**). Conversely, while mean within-system correlations did not exhibit significant age-related differences when examined in aggregate (see **Fig. 2C**, **Fig. S7**), it is clear that several systems exhibit greater within-system correlations in younger versus older mice (red colors in the on-diagonal blocks in the matrices depicted in **Fig. S8**). Notably, two systems in particular show higher within-system RSFC in young adulthood compared with older age: the light blue system ('System 1') comprising medial-frontal brain regions and the beige system ('System 5') comprising retrosplenial and visual brain regions. Interestingly, between-system connections involving these two systems include an exception to the block-level organization of age-related differences. While several sets of between-system relationships involving these two systems are higher in older adulthood, specific edges between anterior cingulate regions and the visual-retrosplenial system are higher in young adulthood, similar to the pattern observed for within-system edges belonging to those two systems. Notably, the circuit connecting anterior cingulate and retrosplenial areas has been proposed as a mouse homologue of the human default mode network (DMN; (58); see also **Fig. S6**).

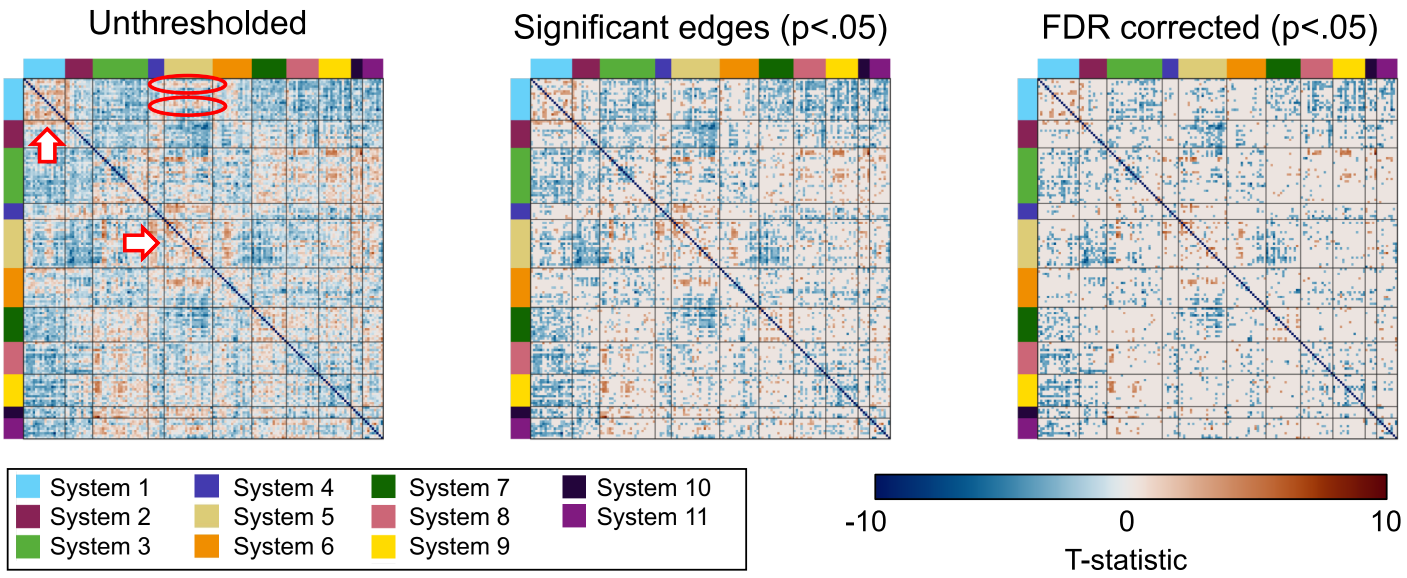

Supplementary Fig. 8. Age-related RSFC network alterations are evident within and between several systems. Edge-level t-statistics based on comparisons between younger (3-4 months) and older (20 months) adult mice are shown for all edges (left), and edges that exhibit statistically reliable differences between groups (p<.05) without (middle) and with FDR correction (right). Negative values reflect lower RSFC in younger adults compared with older adults; positive values reflect higher RSFC in younger adults compared with older adults. Medial-frontal (light blue; 'System 1') and visual-retrosplenial (beige; 'System 5') systems exhibit higher within-system RSFC in younger adults (red arrows). The circuit highlighted in **Fig. S6**, considered an analogue of the human default mode network, is once again apparent (red circles). Despite the anterior cingulate cortex belonging to a different community (light blue) than visual and retrosplenial areas (beige), functional connectivity between these sets of areas is stronger in younger compared to older adults.

2.9 – Age-related declines in system segregation are evident using system labels (communities) defined across a range of edge density values.

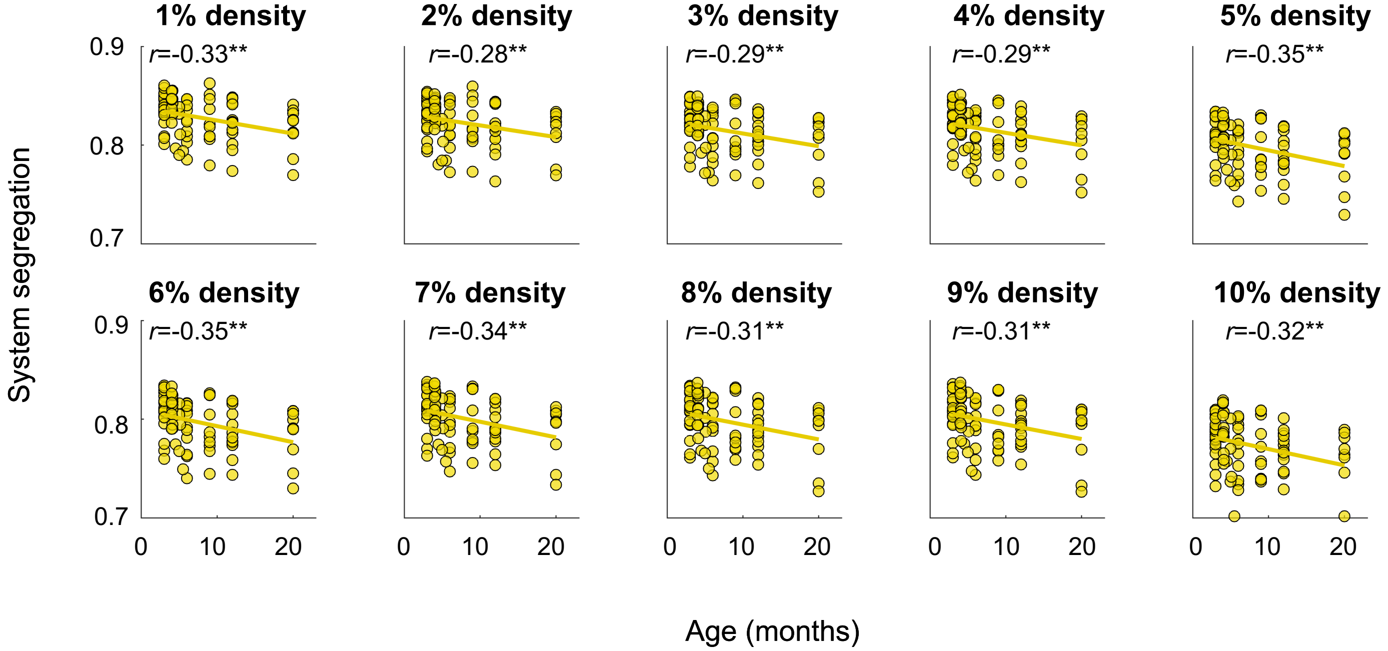

Supplementary Fig 9. Age-related declines in system segregation are evident using system labels (communities) defined across a range of edge density values. System labels (network communities) can be defined at multiple network edge densities. To ensure that the relationship between system segregation and age was not due to the specific edge density used for defining systems used in the primary analysis (7%), relationships were examined from communities defined across a range of edge densities (1-10%, in increments of 1%). Dots depict individual mice. In each of the comparisons above, while edge-density impacts community labels (system labels), system segregation is calculated on the unthresholded correlation matrix (negative edges are set to zero). Statistics (*r*-values) refer to correlation between system segregation and age. $^{**}p<.01$

2.10 – *Age-related declines in system segregation are evident when global signal regression (GSR) is not included in resting-state preprocessing.*

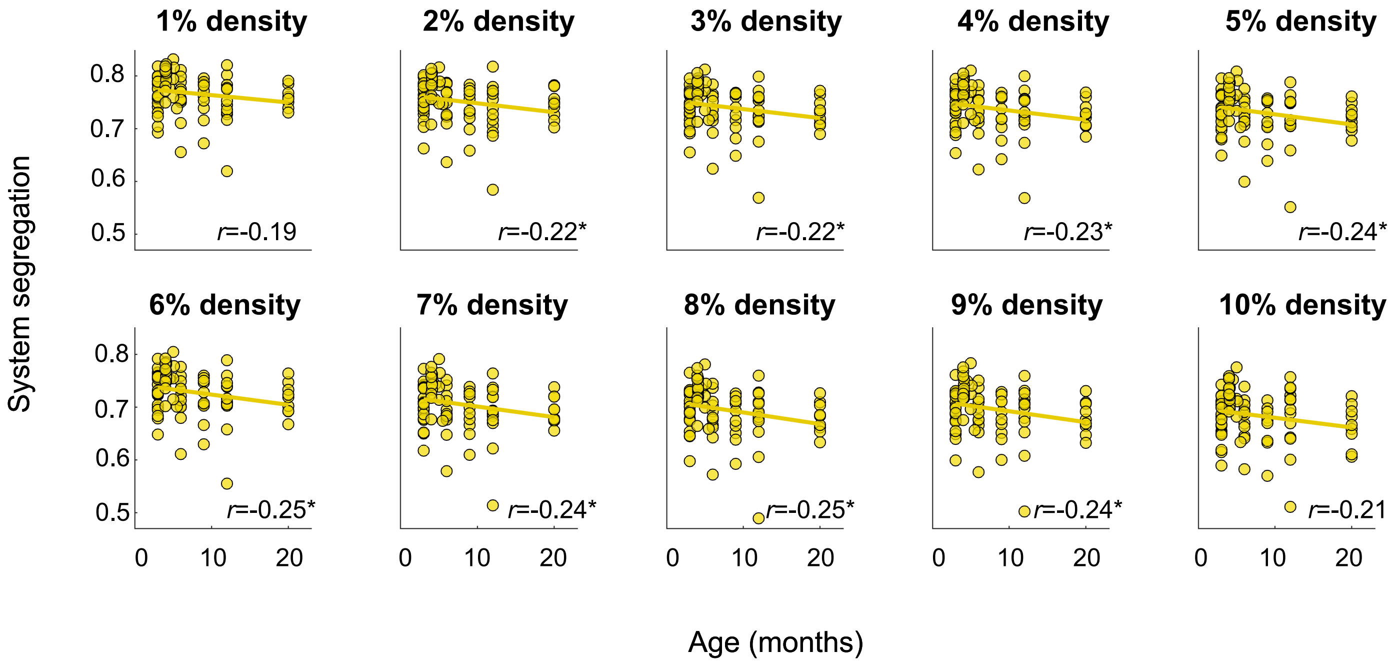

Supplementary Fig 10. Age-related declines in system segregation are evident when global signal regression (GSR) is not included in resting-state preprocessing. Global signal regression was included in the primary mouse preprocessing stream based on its benefits towards minimizing sources of non-neuronal variance and to mirror the preprocessing of human data. To evaluate whether this processing step alters the observed relationship between age and system segregation in mice, mouse data were reprocessed without GSR. As observed when including GSR, mouse system segregation exhibits age-related decline across a range of edge densities. Dots depict individual mice. Statistics (*r*-values) refer to correlation between system segregation and age. *p<.05

2.11 – *Age-related declines in system segregation are evident when using all available clean BOLD data volumes.*

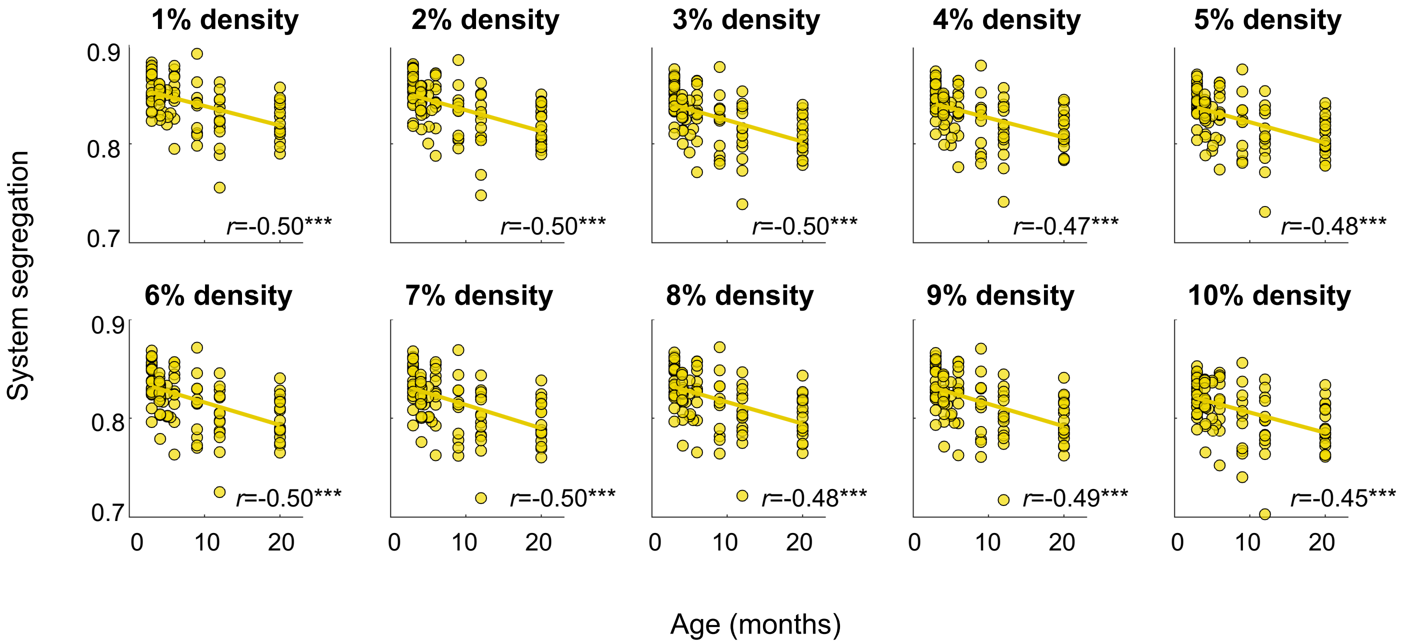

Supplementary Fig 11. Age-related declines in system segregation are evident when using all available clean BOLD data volumes. Even when scan acquisition length is equivalent across mice, the motion-censoring ("scrubbing") step of data preprocessing results in unequal amounts of clean data available per individual. While a common approach is to use all available data to construct RSFC matrices, we have previously demonstrated that using unequal amounts of data can bias cross-subject comparisons of large-scale network organization (20). Accordingly, in the main analysis presented in the paper, system segregation differences and changes are reported using subsampled BOLD timeseries volumes, where equivalent amounts of clean data (volumes) are used across subjects. Here, system segregation is computed using all available clean data for each individual mouse. Across edge densities, system segregation declines with age. Dots depict individual mice. Statistics (*r*-values) refer to correlation between system segregation and age. ***$p<.001$

2.12 – *Age-related declines in system segregation are evident when using network nodes defined from a refined areal parcellation.*

Appropriate node definition is a critical prerequisite for brain network analysis (60, 61). In the context of brain networks, network nodes should represent functionally distinct units of the brain, whereby the nodes are relatively homogenous in terms of their functional properties (at a given spatial resolution (62)), as differences in node homogeneity can bias resultant analyses (28, 63). To test whether the reported results are due to differences in homogeneity across CCFv3 parcels, we created a node atlas consisting of nodes with comparable homogeneity by refining the existing multi-model brain area parcellation (Allen Mouse Brain Atlas, CCFv3; (21)). These nodes were created using mean voxel-level seedmaps from 3 month old mice scanned at the Technion site (n=19, 19 males). Within each of the 156 parcels used in the primary analysis, a 14-voxel ROI (i.e., equal to the minimum parcel size) was derived by finding a functionally representative or "central" voxel, defined as the voxel which had the seedmap most highly correlated with the parcel-level seedmap. Around this voxel, an ROI was grown by selecting the 14 nearest voxels (including the central voxel) within the original parcel boundary. This resulted in a set of 156 equally sized ROIs (**Fig. S12A**); ROIs were not all the same shape due to varying parcel shapes. Following previous work (28, 44), node homogeneity was quantified as the variance explained by the first component of a principal components analysis (PCA) on the voxel-level seedmaps for each node. PCAs were performed separately for each young adult mouse (3-4 months old), and homogeneity values were averaged across mice for each node.

Homogeneity was calculated for each CCFv3 parcel used in the main analysis, as well as each for each parcel-constrained ROI. Parcels defined in the CCFv3 atlas (21) range in size dramatically, and as expected, functional homogeneity calculated on our data varied as a product of parcel size (Pearson's r=-.77, p<.001). In contrast, there was no relationship between the original parcel size and the parcel-constrained ROI homogeneity values (Pearson's r=-.04, p=.63; **Fig. S12B**). In addition, a paired samples t-test revealed higher homogeneity for ROIs than for parcels from which they were derived (t(155)=29.95, p<.001). To test whether the differences in homogeneity between ROIs and parcels were due to differences in size, a null model was created with size- and shape-matched ROIs. For each cortical ROI, 100 spatially permuted null model ROIs were created per mouse by translating the original ROI such that the ROI center fell on the border of two cortical parcels. This analysis was confined to the neocortex, as many subcortical nodes are too small to differentiate between "border" and "non-border" voxels. While this represents a limitation of the null model, cortical and subcortical parcels exhibit similar relationships between size and homogeneity (**Fig. S12B**); it is thus likely that other properties capturing homogeneity, including the comparison with null ROIs, can be extrapolated to the subcortex as well. A paired samples t-test compared homogeneity of the parcel-constrained cortical ROIs with the null-model ROIs, revealing higher homogeneity of the parcel-constrained ROIs (t(77)=45.24, p<.001; **Fig. S12C**).

Finally, system segregation was recalculated based on the parcel-constrained ROIs; across all densities examined, mice exhibited system segregation decline with increasing age (**Fig. S12D**; visualized using system segregation values based on systems derived at 7% edge density [r=-.274, p=.011]). Notably, while this alternate approach to node definition is aimed at addressing one known limitation of the original CCFv3 parcels in terms of application to network construction (i.e., within-node heterogeneity), the ROI approach presents unique limitations of its own. In particular, interindividual variability of RSFC patterns is higher for ROI-based networks than for parcel-based networks. As such, while the relationship between age and system segregation is robust using this alternate node definition which overcomes several limitations of the CCFv3 parcellation, future work should continue to improve upon this approach.

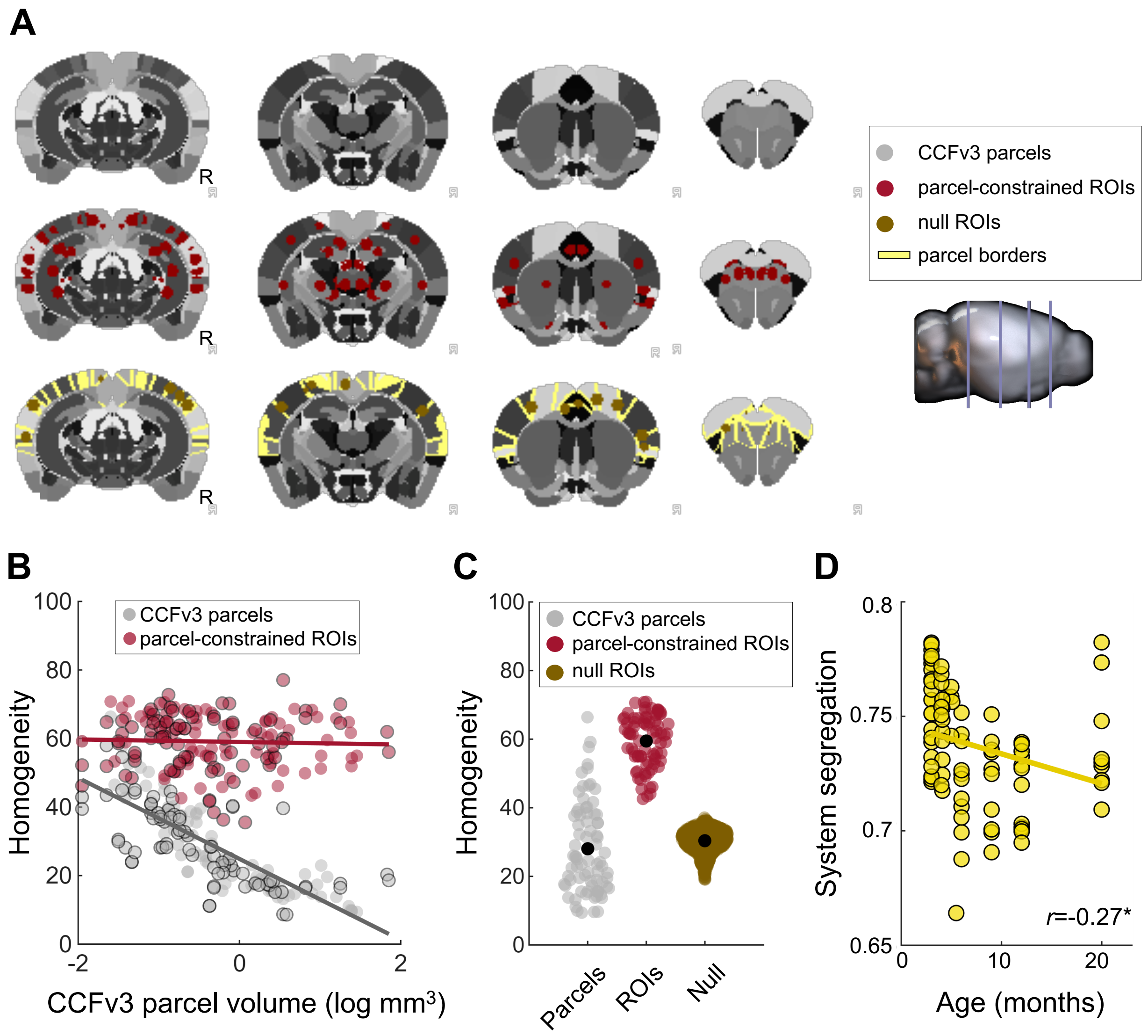

Supplementary Fig. 12. Age-related declines in system segregation are evident when using network nodes defined from a refined areal parcellation. *(A)* Development and evaluation of parcel-constrained functional ROIs. Top row: original Allen Atlas parcels per the CCFv3. Middle row: ROIs were built around the "functional centers" of each parcel, constrained such that they are encompassed by a single parcel. This results in a set of equally sized, but not equally shaped, ROIs. Bottom row: Null model ROIs defined by randomly translating cortical ROIs such that they are centered on cortical parcel boundaries (boundaries in yellow, null ROIs in gold). **(B)** CCFv3 parcels exhibit decreasing homogeneity as parcel size increases, but parcel-constrained ROIs have parcel size-invariant homogeneity. Parcel-constrained ROI homogeneity exceeds parcel homogeneity in both cortical (non-outlined circles) and subcortical regions (outlined). **(C)** Parcel-constrained cortical ROIs have higher homogeneity than both CCFv3 parcels and null ROIs centered on CCFv3 borders, thereby demonstrating that the increased homogeneity of parcel-constrained ROIs is due to homogenous functional signals of constituent voxels rather than spatial autocorrelation. **(D)** System segregation was computed on networks (and corresponding systems) defined with the refined node set (parcel-constrained ROIs). System segregation declines with increasing age; in this plot, system segregation values are based on system labels defined at 7% edge density. Dots depict individual mice. Statistics (*r*-values) refer to correlation between system segregation and age. *p<.05

2.13 – *Age-related system declines in system segregation are evident when using an alternate community detection algorithm.*

The Infomap community detection algorithm was used to identify resting-state brain systems based on previous work which has demonstrated its effectiveness towards this goal (28, 64). Here, an alternate network partitioning algorithm was also used to identify communities (the 'Louvain algorithm'; (65)). As with the procedures involving Infomap community detection procedures, the Louvain community detection algorithm was applied to young adult mouse (3-4 month) RSFC matrices which were first thresholded across a range of densities matching those from the main analysis (i.e., 0.5-20%, in increments of 0.5). The outputs of this algorithm were further processed to yield a final community label set based on the same approach used in the main analysis for Infomap outputs (detailed in Methods and **Fig. S3**). Labels obtained from Louvain and Infomap-based communities share common features, including a community comprising visual and retrosplenial parcels, as well as lateralized communities centered around auditory cortex. Conversely, whereas Infomap-based communities include lateralized somatosensory systems, the Louvain communities merge the somatosensory community with the prefrontal community in the right hemisphere (**Fig. S13A**). NMI between Louvain-based and Infomap-based communities exceeds a null distribution across all densities examined (one-sample t-tests; all $p<.001$; **Fig. S13B**). Modularity (Q) of the young adult matrix (i.e., the matrix used for community detection) was comparable between the two algorithms across all densities (**Fig., S13C**). Importantly, increasing age is associated with decreasing system segregation when the Louvain system labels are applied to the data: consistent with results reported in main text **Fig. 3A** and **Figs. S9-S12**, system segregation derived from Louvain-based labels declines as a function of age, with a similar effect size as communities derived using Infomap (Infomap: r=-.34; Louvain: r=-.32; **Fig. S13D**). Subject-level estimates of system segregation are highly correlated between the Infomap-based and Louvain-based labels (r=.92).

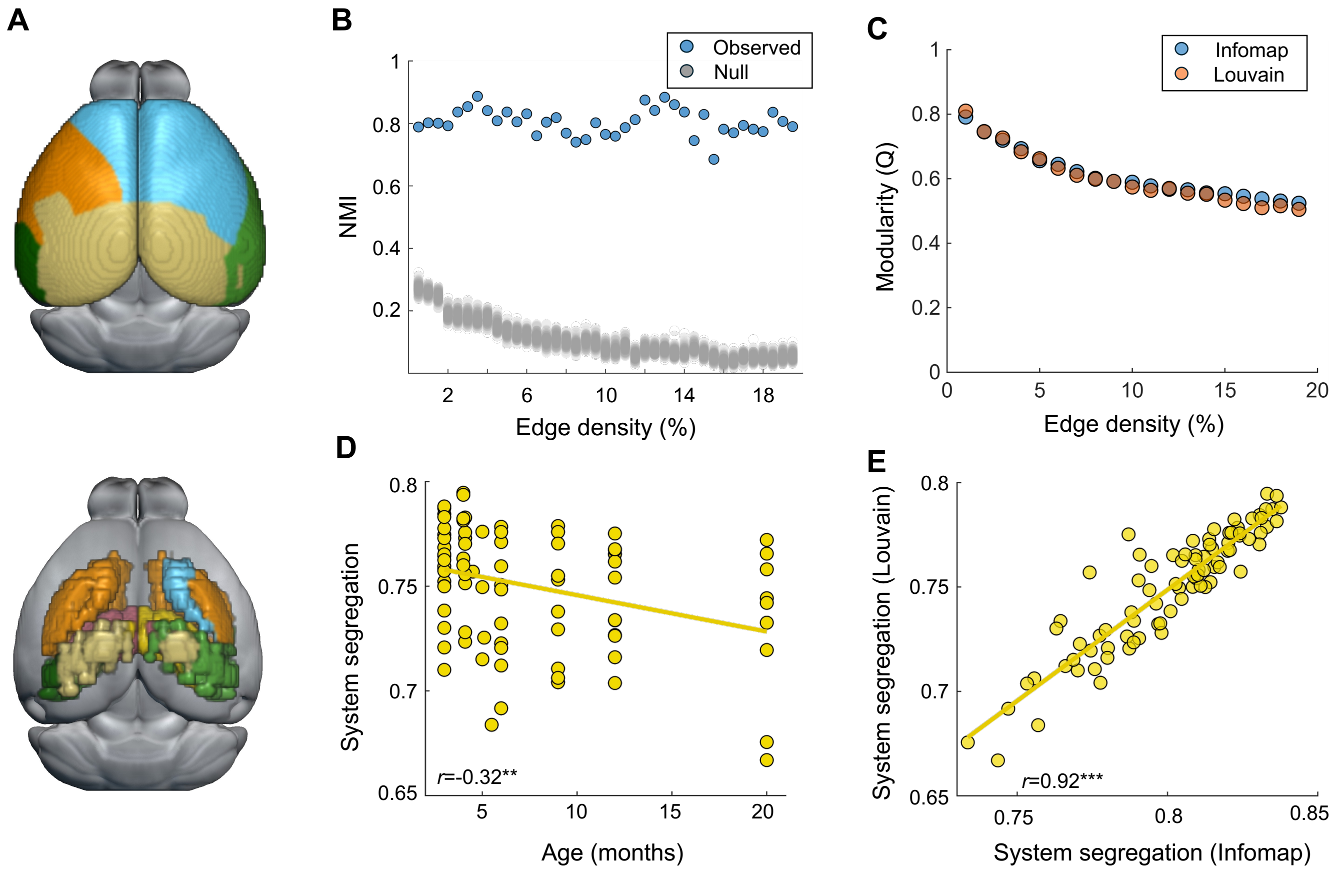

Supplementary Fig 13. Age-related system declines in system segregation are evident when using an alternate community detection algorithm. (A) Depiction of community (system) topography derived from the Louvain algorithm, based on a 7% RSFC network edge density using 3-4 month old mice. Top: cortical parcels.

Bottom: subcortical parcels. **(B)** The community labels obtained from Infomap and Louvain-based communities are similar: The NMI between the two label sets exceeds the NMI of a null distribution across all densities examined. **(C)** Modularity coefficients (Q) of Louvain-based and Infomap-based systems were computed on the young adult group average matrix across a range of edge thresholds. Regardless of threshold, both label sets capture a similar level of modularity. **(D)** Increasing age is associated with decreasing system segregation; in this plot, system labels were obtained using the Louvain-based communities depicted in panel (A), calculated at 7% edge density. **(E)** System segregation values computed using Louvain and Infomap-based communities are highly correlated across animals. ** p<.01, *** p<.001

2.14 – *Longitudinal declines of resting-state system segregation within individual mice during early middle age.*

For a subset of mice, multiple timepoints of data that were acquired from 6-12 months of age were available, allowing for longitudinal data analysis. This age range is approximate to early middle age in mice (i.e., 30-45 years old in humans, though specific age alignments between mice and humans are unresolved; see **Fig. S15**). Given the availability of this individual longitudinal data, to achieve more precise estimates of how individuals' specific network architectures change over time, individual-specific community labels were generated for each mouse based on their network structure at 6 months old. System labels based on 7% edge density were used for each animal, and system segregation was computed using these individual-specific labels for all timepoints that the animal was scanned (i.e., 6 months old, 9 months old, and 12 months old when available). A linear mixed-effects model tested the effect of age on system segregation. The formula used for the linear model was as follows:

system segregation ~ age + (1 + age | individual),

Where individual encodes individual mouse IDs. As all the mice in the longitudinal sample were male, sex was not included in the model.

Increasing age is associated with declines in system segregation ($F(1,23)=10.61$, $p=.003$; **Fig. S14A**). Interestingly, while system segregation in all but one mouse declined over the entire period of time they were scanned (**Fig. S14B**), there existed variability in the magnitude of decline across mice, with some mice declining more over the time period they were scanned and others declining less. This variability parallels aspects of individual differences in brain network changes observed in human aging (66, 67). An important follow-up of the current work will be to identify the factors that contribute to different rates of brain aging and evaluate longitudinal decline across a wider range of the mouse lifespan.

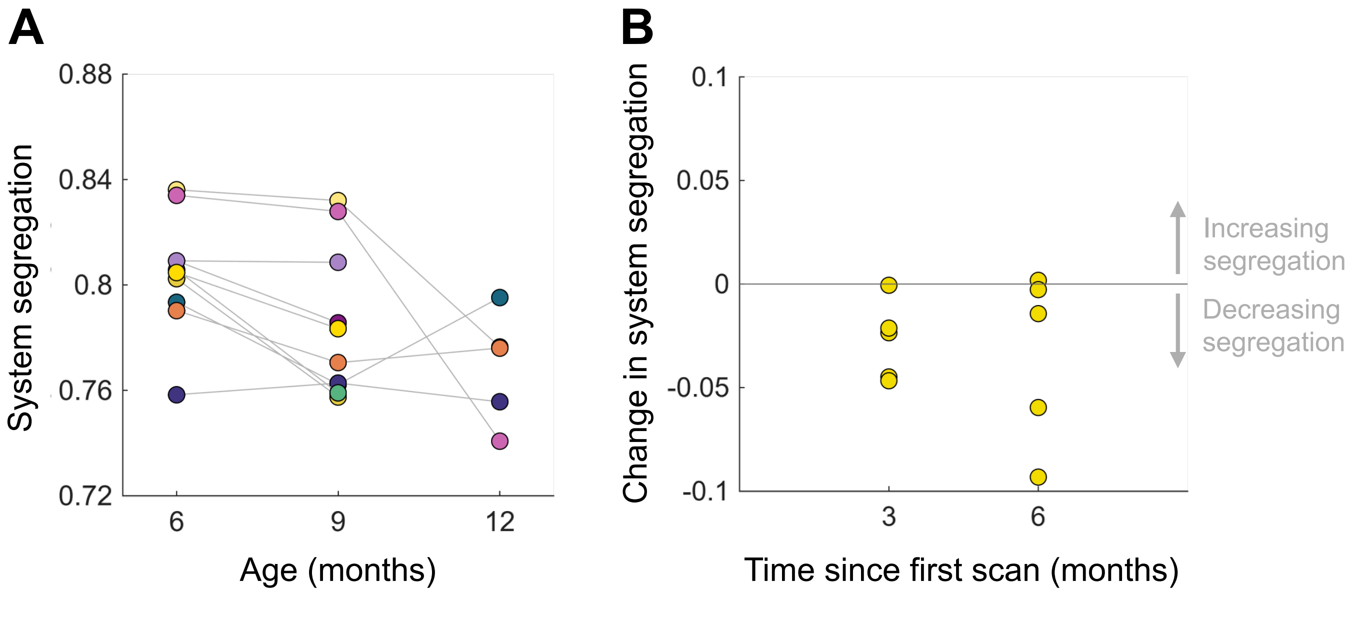

Supplementary Fig 14. Longitudinal declines of resting-state system segregation within individual mice. (A) Across a period of time roughly corresponding to early middle age (6-12 months), system segregation declines longitudinally within individual mice. Dots depict timepoint-specific system segregation values; each color represents an individual mouse, with lines connecting their longitudinal system segregation estimates. **(B)** 9 out of the 10 mice scanned longitudinally exhibited an overall decline in system segregation from the first timepoint to the last timepoint. Dots depict individual mice.

2.15 – *Effects of alternate mouse-human age alignments on cross-species system segregation comparisons.*

Comparison of aging brain network trajectories across species, particularly species with vastly different lifespans, necessitates alignment of ages. Given the present goal of comparing intercepts and slopes of decline, the decision of how to map ages between species directly affects analytic results. For purposes of cross-species comparisons in the present work, we linearly mapped mouse ages, in months, to human ages in years. This approach therefore depended on two variables: the human age considered to be equivalent to the youngest mice in the dataset (i.e., 3 months old) and the human age considered to be equivalent to the oldest mice in the dataset (20 months old). In the primary analysis reported in the main text, we considered a 3 month old mouse to be equivalent to an 18 year old human, and a 20 month old mouse to be equivalent to a 70 year old human, based on converging evidence including life histories, physiology, and cognitive trajectories (49, 68). However, other approaches have characterized 20 month old mice as equivalent to either middle-age or older adult humans, depending on the benchmarking measure (e.g., survival ratio or physical traits, respectively (69); cross-species age mapping may also vary across biological systems (70)). Similarly, 2 month old mice have been considered to be equivalent to humans at 18 years of age, thus making 3 month old mice equivalent to humans in their 20s (70). Accordingly, we repeated the cross-species comparison of system segregation decline across a range of age alignments, independently varying the human ages considered to be equivalent to 3 month old and 20 month old mice. Main effects of age (i.e., system segregation declining with age) and species (i.e., mice having higher system segregation than humans) are present across all alignments tested. The significant age-by-species interaction effect persists across all but the most extreme alignments, demonstrating that the reported cross-species differences are not particular to the specific age alignment used in the main manuscript.

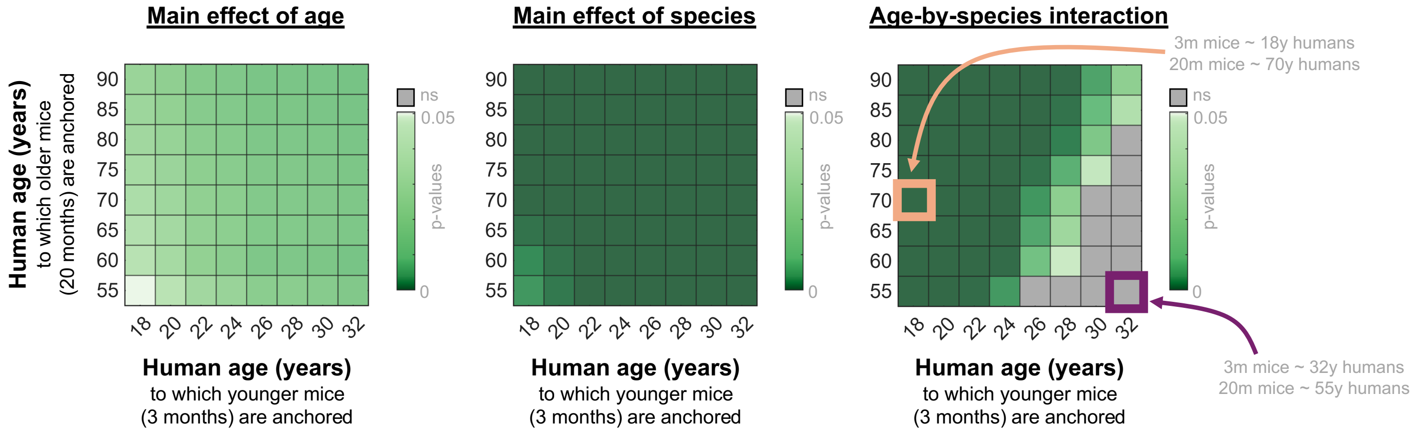

Supplementary Fig 15. Effects of alternate mouse-human age alignments on cross-species system segregation comparisons. Significance testing for the three effects tested in the cross-species model (main effect of age, main effect of species, and age-by-species interaction) for each age alignment. The age-mapping used in the main text is marked with a peach box. Main effects of age and species are present across all alignments tested. For all but a few cross-species age alignments, there exists a significant age-by-species interaction. Only when aligning species based on the extreme and unlikely age alignments (e.g., when 3 month old mice are considered equivalent to 32 year old humans, and 20 month old mice are considered equivalent to 55 year old humans; purple box) do the two species show more similar rates of system segregation decline. Colormap represents *p*-values for the various effects; darker greens reflect *p*-values approaching zero. Instances where *p*>.05 (i.e., non-significant) are shown in gray.

2.16 – *Comparison of mouse and human system segregation trajectories in the absence of global signal regression (GSR)*

Global signal regression (GSR) was included in the primary preprocessing pipeline based on its documented benefits in reducing spurious resting-state correlations arising from motion and physiological noise (32–34). To evaluate the robustness of our findings to this preprocessing choice, a secondary analysis computed system segregation in mice and humans using data in which the global signal was retained. This analysis yielded results that were consistent with those of the primary pipeline. As shown in **Fig. S10**, increasing age was associated with declining system segregation in mice even when the global signal was preserved during preprocessing (one mouse observation included in the primary analysis was identified as a statistical outlier and excluded from the comparisons detailed below).

As expected, retaining the global signal resulted in overall lower system segregation values in mice relative to GSR-based estimates (**Fig. S16A**), while age-related declines in system segregation remained evident (r=-0.219, p=.045). Comparable effects were observed in human data: age-related declines in system segregation were also present when the global signal was retained (r=-0.234, p<.001), although segregation values were lower relative to GSR-based estimates (**Fig. S16B**).

Replicating the analysis reported in **Fig. 3C**, non-GSR-based mouse and human system segregation values were directly compared in a linear mixed effects model (**Fig. S16C)**. Paralleling the main analysis, a robust main effect of species was observed (F(1,1186) = 498.56, *p*<.001), such that system segregation is higher in mice than in humans. In contrast to the GSR-based analysis, no main effect of age was detected when species were combined (F(1,1186)=0.015, *p*=.903), although there was a significant age-by-species interaction (F(1,1186)=4.698, *p*=.030), such that humans exhibit greater age-related declines in system segregation than mice (mouse β: -0.063, human β: -0.183; β values come from the cross-species model).

Importantly, non-GSR system segregation estimates exhibited substantially greater variance than GSR-based estimates (standard deviations: $\text{mouse}_{\text{GSR}}$=0.025, $\text{mouse}_{\text{non-GSR}}$=0.040, $\text{human}_{\text{GSR}}$=0.042, $\text{human}_{\text{non-GSR}}$=0.104), likely reflecting increased contributions from motion and physiological noise and resulting in reduced statistical sensitivity in the combined-species model. Consistent with this interpretation, **Fig. S16A–B** demonstrates that age-related trajectories of system segregation are qualitatively similar and roughly parallel between GSR-based and non-GSR-based analyses within each species. Thus, the reduced statistical support for age effects in the combined-species model is most parsimoniously attributed to increased noise rather than meaningful differences in age-related patterns of network organization.

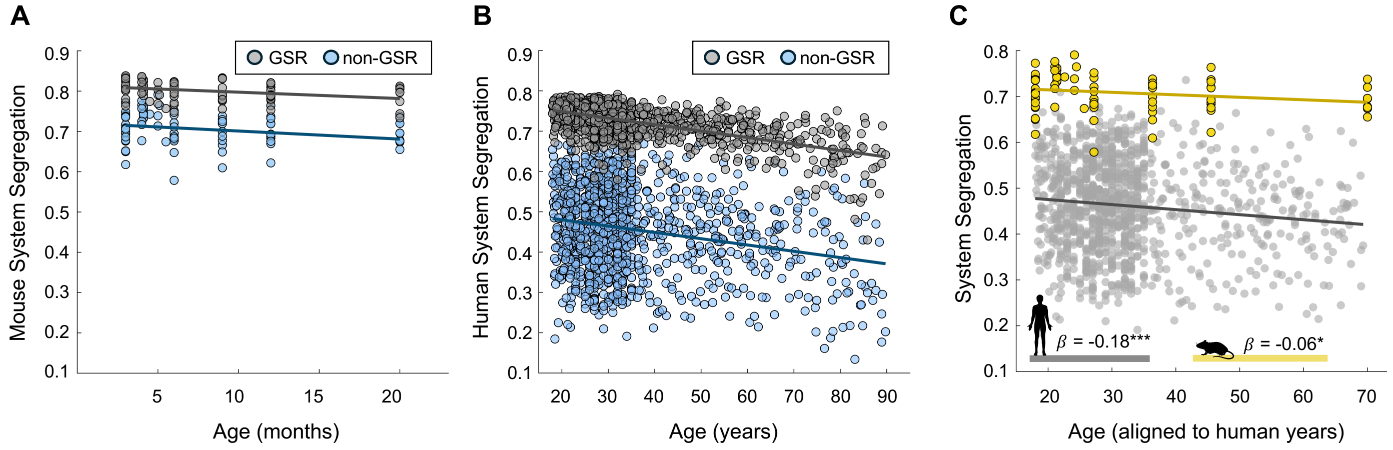

Supplementary Fig 16. **Comparison of cross-species system segregation trajectories in the absence of global signal regression (GSR). (A)** In mice, GSR results in higher system segregation values compared with the analysis in which the global signal is retained (non-GSR). System segregation declines with age under both preprocessing streams (GSR: *r*=-0.343, *p*=.001; nonGSR: *r*=-0.219, *p*=.045), but variance is noticeably higher in the non-GSR estimates (SD_{GSR}=0.025; $\text{SD}_{\text{nonGSR}}$=0.040). Mouse system segregation values are based on system

labels defined at 7% edge density. **(B)** Comparable effects of GSR are observed in humans: system segregation declines with age in both preprocessing streams (GSR: r=-0.608, p<.001; nonGSR: r=-0.235, p<.001), while retaining the global signal results in lower system segregation values and increased interindividual variability (SD_{GSR}=0.042; SD_{nonGSR}=0.104). **(C)** Consistent with the GSR-based analysis, cross-species comparison using non-GSR-based data reveal a main effect of species, with higher system segregation in mice than in humans across ages, and a steeper age-related decline in humans than in mice. β values listed in the figure panel come from the cross-species model. *p<.05; ***p<.001

2.17 – *Cross-species differences in system segregation reflect reduced long-range integration of RSFC systems in older age mice.*

Analyses examining the relationship between connection distances and between-system RSFC (**Fig. 4**) were repeated using older adult mice (20 months old) and humans (70-90 years old; **Fig. S17**). As in the primary analysis, the group average matrix was used for each species. A 2×2 ANOVA assessed RSFC strength as a function of species and connection type (within- vs. between-system edges; **Fig. S17A-B**), revealing significant main effects of species ($F(1,50951)=454.23$, $p<.001$) and connection type ($F(1,50951)=10507.34$, $p<.001$), as well as a species-by-connection type interaction ($F(1,50951)=1113.90$, $p<.001$). Consistent with the young adult analysis, within-system RSFC is higher than between-system for both species, but the difference between within-system and between-system RSFC is greater for mice, contributing to their higher overall system segregation. We classified between-system RSFC edges as short- or long-range based on Euclidean distance between regions within each species. A 2×2 ANOVA on RSFC strength across these distance-defined RSFC categories (**Fig. S17C-D**) revealed main effects of species ($F(1,45297)=418.66$, $p<.001$) and connection distance ($F(1,45297)=2054.34$, $p<.001$), as well as a species-by-distance interaction ($F(1,45297)=126.99$, $p<.001$). Mice exhibited reduced RSFC among long-range between-system relationships relative to short-range relationships, compared to humans, who showed less distinct distributions of long- versus short-range between-system RSFC.

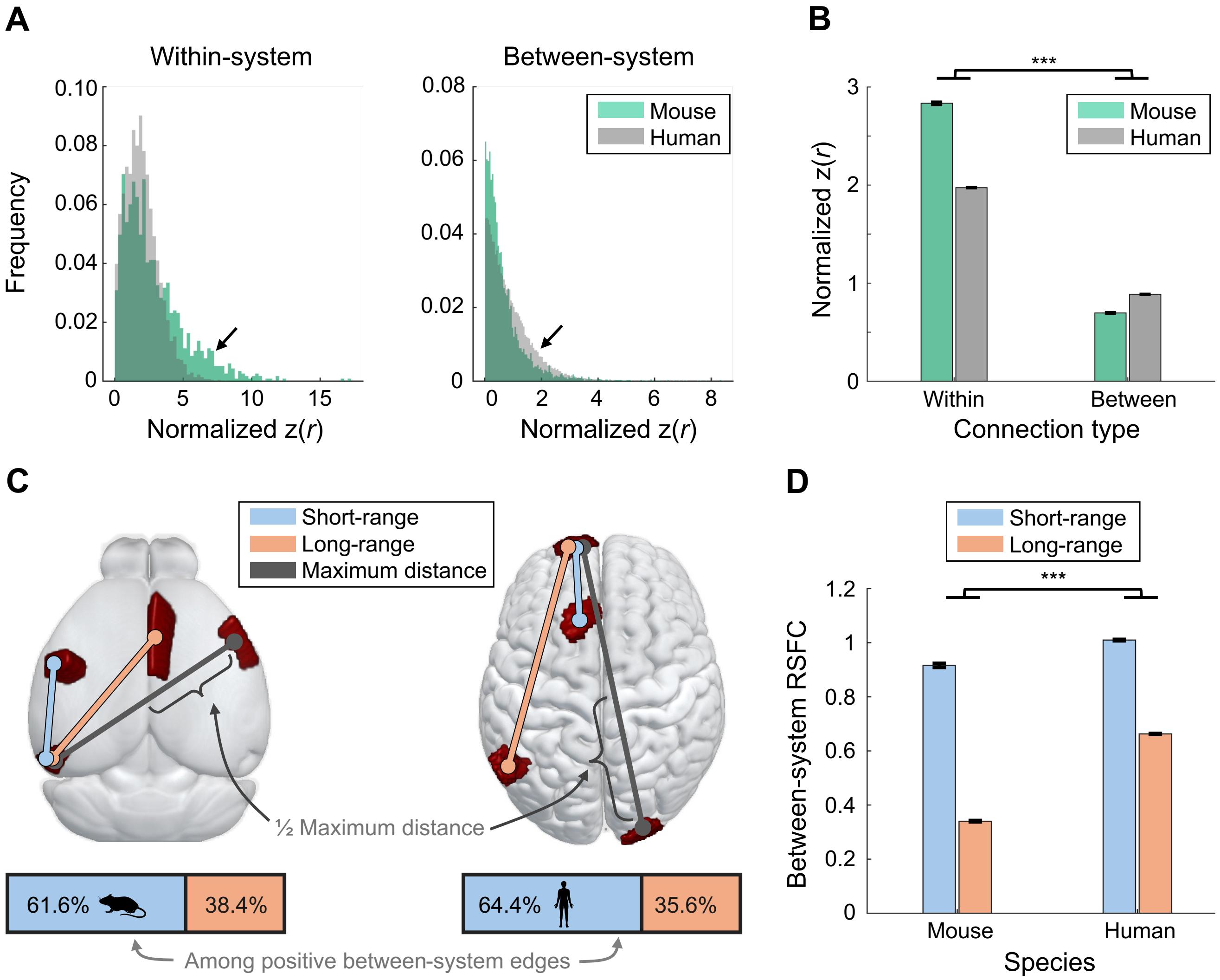

Supplementary Fig 17. Cross-species differences in system segregation reflect reduced long-range integration of RSFC systems in older age mice. (A) Among older adults, mice show stronger within-system RSFC than humans, while humans exhibit stronger between-system RSFC than mice. Arrows highlight these

differences between species. **(B)** Although within-system RSFC exceeds between-system RSFC in both species, the contrast is significantly greater in mice, leading to higher system segregation. Error bars represent standard error. **(C)** Between-system edges were categorized according to the physical distance between each pair of regions (short-range versus long range, based on whether the distance was shorter or longer than half the maximum distance of region-to-region relationships measured for that species). Horizontal stacked bars below depict proportions of functional connection types. **(D)** The distance-based distinction is also evident from comparisons of RSFC strength. Bar plots depict mean RSFC strength of short-range versus long-range between-system relationships: mice show a greater difference in functional connectivity strength in relation to between-system relationship distance, compared to humans. Error bars represent standard error. ***p<.001.

2.18 – *List of injections used for definition of striatal sub-regions*

Previous investigations show that the CCFv3 Caudoputamen parcel can be subdivided based on anatomical connectivity patterns, and that these subdivisions exhibit distinct functional connectivity (22). Normalized intensity data from the listed tracing experiments were used to further divide the Caudoputamen area into 6 subdivisions based on a winner-take all approach, as detailed in the Methods.

Injection #	Subregion assignment	Subregion name
120437703	1	Auditory
100149109	1	Auditory
112881858	1	Auditory
112458114	2	Frontal
126860974	2	Frontal
157711748	2	Frontal
112596790	3	Lateral
100148142	4	Medial
112424813	4	Medial
100148503	4	Medial
112951804	5	Somatomotor
113036264	5	Somatomotor
100141780	5	Somatomotor
100141599	6	Visual
277616630	6	Visual
100147853	6	Visual

Table S1. Injections used for definition of striatal sub-regions using normalized intensity.

3. SUPPLEMENTARY REFERENCES

1. D. C. Van Essen, *et al.*, The WU-Minn Human Connectome Project: an overview. *NeuroImage* **80**, 62–79 (2013).
2. S. Y. Bookheimer, *et al.*, The Lifespan Human Connectome Project in Aging: An overview. *NeuroImage* **185**, 335–348 (2019).
3. L. H. Somerville, *et al.*, The Lifespan Human Connectome Project in Development: A large-scale study of brain connectivity development in 5–21 year olds. *NeuroImage* **183**, 456–468 (2018).
4. E. Bergmann, G. Zur, G. Bershadsky, I. Kahn, The Organization of Mouse and Human Cortico-Hippocampal Networks Estimated by Intrinsic Functional Connectivity. *Cereb. Cortex N. Y. N 1991* **26**, 4497–4512 (2016).
5. J. Asleh, *et al.*, Brain-wide structural and functional disruption in mice with oligodendrocyte-specific Nf1 deletion is rescued by inhibition of nitric oxide synthase. *Proc. Natl. Acad. Sci. U. S. A.* **117**, 22506–22513 (2020).
6. E. Bergmann, X. Gofman, A. Kavushansky, I. Kahn, Individual variability in functional connectivity architecture of the mouse brain. *Commun. Biol.* **3**, 1–10 (2020).
7. D. Lichtman, *et al.*, Structural and functional brain-wide alterations in A350V Iqsec2 mutant mice displaying autistic-like behavior. *Transl. Psychiatry* **11**, 1–13 (2021).
8. B. Shofty, *et al.*, Autism-associated Nf1 deficiency disrupts corticocortical and corticostriatal functional connectivity in human and mouse. *Neurobiol. Dis.* **130**, 104479 (2019).
9. R. W. Chan, *et al.*, NOise Reduction with DIstribution Corrected (NORDIC) principal component analysis improves brain activity detection across rodent and human functional MRI contexts. *Imaging Neurosci.* **2**, imag–2–00325 (2024).
10. L. Vizioli, *et al.*, Lowering the thermal noise barrier in functional brain mapping with magnetic resonance imaging. *Nat. Commun.* **12**, 5181 (2021).
11. R. W. Chan, *et al.*, NOise Reduction with DIstribution Corrected (NORDIC) PCA improves signal-to-noise in rodent resting-state and optogenetic functional MRI in *2022 44th Annual International Conference of the IEEE Engineering in Medicine & Biology Society (EMBC)*, (2022), pp. 1847–1850.
12. T. T. Liu, Noise contributions to the fMRI signal: An overview. *NeuroImage* **143**, 141–151 (2016).
13. L. Vizioli, *et al.*, Lowering the thermal noise barrier in functional brain mapping with magnetic resonance imaging. *Nat. Commun.* **12**, 5181 (2021).
14. G. Desrosiers-Grégoire, G. A. Devenyi, J. Grandjean, M. M. Chakravarty, A standardized image processing and data quality platform for rodent fMRI. *Nat. Commun.* **15**, 6708 (2024).
15. A. E. Dorr, J. P. Lerch, S. Spring, N. Kabani, R. M. Henkelman, High resolution three-dimensional brain atlas using an average magnetic resonance image of 40 adult C57Bl/6J mice. *NeuroImage* **42**, 60–69 (2008).
16. P. E. Steadman, *et al.*, Genetic effects on cerebellar structure across mouse models of autism using a magnetic resonance imaging atlas. *Autism Res. Off. J. Int. Soc. Autism Res.* **7**, 124–137 (2014).
17. K. Richards, *et al.*, Segmentation of the mouse hippocampal formation in magnetic resonance images. *NeuroImage* **58**, 732–740 (2011).
18. J. F. P. Ullmann, C. Watson, A. L. Janke, N. D. Kurniawan, D. C. Reutens, A segmentation protocol and MRI atlas of the C57BL/6J mouse neocortex. *NeuroImage* **78**, 196–203 (2013).
19. J. D. Power, *et al.*, Methods to detect, characterize, and remove motion artifact in resting state fMRI. *NeuroImage* **84**, 10.1016/j.neuroimage.2013.08.048 (2014).
20. L. Han, *et al.*, Measures of resting-state brain network segregation and integration vary in relation to data quantity: implications for within and between subject comparisons of functional brain network organization. *Cereb. Cortex* **34**, bhad506 (2024).
21. Q. Wang, *et al.*, The Allen Mouse Brain Common Coordinate Framework: A 3D Reference Atlas. *Cell* **181**, 936-953.e20 (2020).
22. J. Grandjean, V. Zerbi, J. H. Balsters, N. Wenderoth, M. Rudin, Structural Basis of Large-Scale Functional Connectivity in the Mouse. *J. Neurosci. Off. J. Soc. Neurosci.* **37**, 8092–8101 (2017).

23. A. A. Chen, *et al.*, Mitigating site effects in covariance for machine learning in neuroimaging data. *Hum. Brain Mapp.* **43**, 1179–1195 (2022).
24. M. Rosvall, C. T. Bergstrom, Maps of random walks on complex networks reveal community structure. *Proc. Natl. Acad. Sci.* **105**, 1118–1123 (2008).
25. S. Marek, *et al.*, Spatial and Temporal Organization of the Individual Human Cerebellum. *Neuron* **100**, 977-993.e7 (2018).
26. B. A. Seitzman, *et al.*, A set of functionally-defined brain regions with improved representation of the subcortex and cerebellum. *NeuroImage* **206**, 116290 (2020).
27. J. D. Power, *et al.*, Functional network organization of the human brain. *Neuron* **72**, 665–678 (2011).
28. E. M. Gordon, *et al.*, Generation and Evaluation of a Cortical Area Parcellation from Resting-State Correlations. *Cereb. Cortex N. Y. N 1991* **26**, 288–303 (2016).
29. M. F. Glasser, *et al.*, The minimal preprocessing pipelines for the Human Connectome Project. *NeuroImage* **80**, 105–124 (2013).
30. D. S. Marcus, *et al.*, Human Connectome Project informatics: Quality control, database services, and data visualization. *NeuroImage* **80**, 202–219 (2013).
31. D. C. Van Essen, M. F. Glasser, D. L. Dierker, J. Harwell, T. Coalson, Parcellations and Hemispheric Asymmetries of Human Cerebral Cortex Analyzed on Surface-Based Atlases. *Cereb. Cortex* **22**, 2241–2262 (2012).
32. F. M. Miezin, L. Maccotta, J. M. Ollinger, S. E. Petersen, R. L. Buckner, Characterizing the Hemodynamic Response: Effects of Presentation Rate, Sampling Procedure, and the Possibility of Ordering Brain Activity Based on Relative Timing. *NeuroImage* **11**, 735–759 (2000).
33. J. D. Power, B. L. Schlaggar, S. E. Petersen, Studying Brain Organization via Spontaneous fMRI Signal. *Neuron* **84**, 681–696 (2014).
34. J. D. Power, M. Plitt, T. O. Laumann, A. Martin, Sources and implications of whole-brain fMRI signals in humans. *NeuroImage* **146**, 609–625 (2017).
35. T. D. Satterthwaite, *et al.*, An improved framework for confound regression and filtering for control of motion artifact in the preprocessing of resting-state functional connectivity data. *NeuroImage* **64**, 240–256 (2013).
36. J. D. Power, *et al.*, Ridding fMRI data of motion-related influences: Removal of signals with distinct spatial and physical bases in multiecho data. *Proc. Natl. Acad. Sci.* **115**, E2105–E2114 (2018).
37. N. K. Savalia, *et al.*, Motion-related artifacts in structural brain images revealed with independent estimates of in-scanner head motion. *Hum. Brain Mapp.* **38**, 472–492 (2017).
38. K. R. A. Van Dijk, M. R. Sabuncu, R. L. Buckner, The influence of head motion on intrinsic functional connectivity MRI. *NeuroImage* **59**, 431–438 (2012).
39. D. A. Fair, *et al.*, Correction of respiratory artifacts in MRI head motion estimates. *NeuroImage* **208**, 116400 (2020).
40. A. Schaefer, *et al.*, Local-Global Parcellation of the Human Cerebral Cortex from Intrinsic Functional Connectivity MRI. *Cereb. Cortex N. Y. NY* **28**, 3095–3114 (2018).
41. R. Kong, *et al.*, Individual-Specific Areal-Level Parcellations Improve Functional Connectivity Prediction of Behavior. *Cereb. Cortex* **31**, 4477–4500 (2021).
42. M. Y. Chan, D. C. Park, N. K. Savalia, S. E. Petersen, G. S. Wig, Decreased segregation of brain systems across the healthy adult lifespan. *Proc. Natl. Acad. Sci. U. S. A.* **111**, E4997-5006 (2014).
43. Z. Zhang, *et al.*, Dissociable Effects of Alzheimer's Disease-Related Cognitive Dysfunction and Aging on Functional Brain Network Segregation. *J. Neurosci. Off. J. Soc. Neurosci.* **43**, 7879–7892 (2023).
44. L. Han, *et al.*, Functional Parcellation of the Cerebral Cortex Across the Human Adult Lifespan. *Cereb. Cortex N. Y. NY* **28**, 4403–4423 (2018).
45. K. Murphy, M. D. Fox, Towards a consensus regarding global signal regression for resting state functional connectivity MRI. *NeuroImage* **154**, 169–173 (2017).
46. M. L. Schölvinck, A. Maier, F. Q. Ye, J. H. Duyn, D. A. Leopold, Neural basis of global resting-state fMRI activity. *Proc. Natl. Acad. Sci.* **107**, 10238–10243 (2010).
47. L. Engqvist, The mistreatment of covariate interaction terms in linear model analyses of behavioural and evolutionary ecology studies. *Anim. Behav.* **70**, 967–971 (2005).

48. N. C. Cottam, *et al.*, From circuits to lifespan: translating mouse and human timelines with neuroimaging based tractography. [Preprint] (2024). Available at: http://biorxiv.org/lookup/doi/10.1101/2024.07.28.605528 [Accessed 8 August 2024].
49. K. Flurkey, J. Currer, D. Harrison, The Mouse in Aging Research. *Mouse Biomed. Res.* **3** (2007).
50. J. Grandjean, *et al.*, Common functional networks in the mouse brain revealed by multi-centre resting-state fMRI analysis. *NeuroImage* **205**, 116278 (2020).
51. E. G. Jones, J. D. Coulter, S. P. Wise, Commissural columns in the sensory-motor cortex of monkeys. *J. Comp. Neurol.* **188**, 113–135 (1979).
52. B. Biswal, F. Z. Yetkin, V. M. Haughton, J. S. Hyde, Functional connectivity in the motor cortex of resting human brain using echo-planar MRI. *Magn. Reson. Med.* **34**, 537–541 (1995).
53. J. L. Vincent, *et al.*, Intrinsic functional architecture in the anaesthetized monkey brain. *Nature* **447**, 83–86 (2007).
54. F. Sforazzini, A. J. Schwarz, A. Galbusera, A. Bifone, A. Gozzi, Distributed BOLD and CBV-weighted resting-state networks in the mouse brain. *NeuroImage* **87**, 403–415 (2014).
55. J. A. Harris, *et al.*, Hierarchical organization of cortical and thalamic connectivity. *Nature* **575**, 195–202 (2019).
56. M. E. J. Newman, M. Girvan, Finding and evaluating community structure in networks. *Phys. Rev. E* **69**, 026113 (2004).
57. V. Zerbi, J. Grandjean, M. Rudin, N. Wenderoth, Mapping the mouse brain with rs-fMRI: An optimized pipeline for functional network identification. *NeuroImage* **123**, 11–21 (2015).
58. A. Gozzi, A. J. Schwarz, Large-scale functional connectivity networks in the rodent brain. *NeuroImage* **127**, 496–509 (2016).
59. J. D. Whitesell, *et al.*, Regional, Layer, and Cell-Type-Specific Connectivity of the Mouse Default Mode Network. *Neuron* **109**, 545-559.e8 (2021).
60. G. S. Wig, B. L. Schlaggar, S. E. Petersen, Concepts and principles in the analysis of brain networks. *Ann. N. Y. Acad. Sci.* **1224**, 126–146 (2011).
61. A. Zalesky, *et al.*, Whole-brain anatomical networks: does the choice of nodes matter? *NeuroImage* **50**, 970–983 (2010).
62. S. E. Petersen, B. A. Seitzman, S. M. Nelson, G. S. Wig, E. M. Gordon, Principles of cortical areas and their implications for neuroimaging. *Neuron* (2024). https://doi.org/10.1016/j.neuron.2024.05.008.
63. T. Xu, *et al.*, Delineating the Macroscale Areal Organization of the Macaque Cortex *In Vivo*. *Cell Rep.* **23**, 429–441 (2018).
64. J. D. Power, *et al.*, Functional Network Organization of the Human Brain. *Neuron* **72**, 665–678 (2011).
65. V. D. Blondel, J.-L. Guillaume, R. Lambiotte, E. Lefebvre, Fast unfolding of communities in large networks. *J. Stat. Mech. Theory Exp.* **2008**, P10008 (2008).
66. M. Y. Chan, *et al.*, Long-term prognosis and educational determinants of brain network decline in older adult individuals. *Nat. Aging* **1**, 1053–1067 (2021).
67. J. S. X. Chong, *et al.*, Longitudinal Changes in the Cerebral Cortex Functional Organization of Healthy Elderly. *J. Neurosci.* **39**, 5534–5550 (2019).
68. S. Dutta, P. Sengupta, Men and mice: Relating their ages. *Life Sci.* **152**, 244–248 (2016).
69. S. Yanai, S. Endo, Functional Aging in Male C57BL/6J Mice Across the Life-Span: A Systematic Behavioral Analysis of Motor, Emotional, and Memory Function to Define an Aging Phenotype. *Front. Aging Neurosci.* **13**, 697621 (2021).
70. N. Geifman, E. Rubin, The mouse age phenome knowledgebase and disease-specific inter-species age mapping. *PloS One* **8**, e81114 (2013).
